# R-spondins suppress adipogenic differentiation in fibro-adipogenic progenitors via autocrine WNT/β-catenin signaling

**DOI:** 10.3389/fcell.2026.1942626

**Published:** 2026-10-16

**Authors:** Jennifer Fransisca, Younjeong Oh, Bagus Sarmito, Nurkyz Alymkulova, Yongsung Hwang, Jeong Kyo Yoon

**Affiliations:** 1 Department of Integrated Biomedical Science, Graduate School, Soonchunhyang University, Cheonan, Republic of Korea; 2 Soonchunhyang Institute of Medi-Bio Science, Soonchunhyang University, Cheonan, Republic of Korea

**Keywords:** adipogenic differentiation, fibro-adipogenic progenitors, R-spondins, skeletal muscle, Wnt/β-catenin signaling

## Abstract

**Introduction:**

Fibro‐adipogenic progenitors (FAPs) are muscle‐resident stromal cells that support skeletal muscle regeneration and homeostasis but can drive pathological adipogenesis. In this study, we investigated the role of R‐spondins (RSPOs), a family of WNT signaling modulators, in FAP adipogenic differentiation.

**Methods:**

FAPs were differentiated in adipogenic induction medium (AIM) supplemented with RSPO2 or RSPO3 in combination with WNT3A, followed by assessment of adipogenic differentiation using western blot and Oil Red O (ORO) analyses. siRNA‐mediated knockdown of *Rspo*2, *Rspo*3, and *Ctnnb1*, as well as constitutive activation of β-catenin, was performed to investigate their roles in FAP adipogenesis. *For in vivo* validation, a mixture of recombinant RSPO3 and WNT3A was injected into glycerol‐injured tibialis anterior muscles, followed by assessment of adipogenesis and transcriptomic analysis.

**Results:**

Here, we identify *Rspo*2 and *Rspo*3 as key autocrine regulators of FAP fate through modulation of WNT/β‐catenin signaling. Their siRNA‐mediated knockdown markedly enhances FAP adipogenic differentiation. Conversely, RSPO2 and RSPO3 synergize with WNT3A to suppress FAP adipogenesis, an effect dependent on β‐catenin as demonstrated by attenuation following *Ctnnb1* knockdown. Constitutive β‐catenin activation similarly restrains adipogenic differentiation. *In vivo*, intramuscular delivery of recombinant RSPO3 and WNT3A proteins significantly reduces ectopic adipogenesis induced in glycerol‐injured tibialis anterior muscle, accompanied by downregulation of adipogenic markers and transcriptional programs.

**Discussion:**

Collectively, these findings establish RSPO2 and RSPO3 as previously unrecognized suppressors of FAP adipogenesis acting through canonical WNT/β‐catenin signaling.

## 1 Introduction

Fibro-adipogenic progenitors (FAPs) are a heterogenous population of mesenchymal stromal cells residing in the interstitium of skeletal muscle, characterized by the expression of stem cell antigen-1 (SCA-1), CD34, and platelet-derived growth factor receptor alpha (PDGFRα) (Contreras et al., 2021; Flores-Opazo et al., 2024). They function as a critical cellular niche that supports neighboring cells, including skeletal muscle stem cells (MuSCs) and myofibers (Biferali et al., 2019; Hogarth et al., 2025). Their genetic ablation leads to impaired muscle mass regulation and regeneration (Wosczyna et al., 2019), highlighting their essential role as a paracrine source for muscle homeostasis.

In addition, owing to their capacity to differentiate into multiple lineages, including adipocytes and fibroblasts (Theret et al., 2021), FAPs are key regulators of muscle integrity under both physiological and pathological conditions. In disease states, FAPs contribute to pathological remodeling of muscle, including myosteatosis and myofibrosis observed in degenerative muscle disorders such as Duchenne muscular dystrophy (DMD) (Cardone et al., 2023) and aging muscle (Moratal et al., 2019). FAPs-derived myofibrosis has also been implicated in functional decline of neuromuscular junctions (NMJs) in acute spinal cord injury and NMJ-associated motor neuron diseases (Madaro et al., 2018). Therefore, understanding the regulatory mechanisms governing FAP differentiation is essential, and accumulating evidence indicates multiple signaling pathways, including NOTCH (Marinkovic et al., 2019), Hedgehog (Kopinke et al., 2017), interleukin-mediated signaling (Heredia et al., 2013; Kang et al., 2018), and WNT signaling (Reggio et al., 2020; Fu et al., 2023), play crucial roles in modulating FAP fate.

R-spondins (RSPOs) are a family of secreted cysteine-rich glycoproteins within the thrombospondin type 1 repeat-containing protein superfamily, comprising four members (RSPO1-4) that share conserved structural features, including two adjacent furin-like cysteine-rich domains (FU-CRD1/2), a thrombospondin type I repeat (TSR) domain, and a variable C-terminal basic amino acid (BR) domain (Jin and Yoon, 2012; Raslan and Yoon, 2019). Their best characterized function is to potentiate canonical WNT signaling by binding leucine-rich repeat containing G protein-coupled receptors 4/5/6 (LGR4/5/6) and the plasma membrane-bound E3 ubiquitin ligases ring finger 3 (ZNRF3) and ring finger protein 43 (RNF43), thereby stabilizing WNT receptors at plasma membrane (Chen et al., 2013; Wang et al., 2013; Zebisch et al., 2013). Alternatively, RSPOs can potentiate WNT signaling through syndecan/heparan sulfate proteoglycan-dependent mechanisms involving the TSR/BR domains (Dubey et al., 2020; Lebensohn et al., 2022). Recently, both RSPO2 and RSPO3 have been reported to inhibit adipogenic differentiation in adipose progenitors (Loh et al., 2020; Dong et al., 2022; Reed et al., 2024). However, RSPO-mediated signaling in FAPs remains poorly understood, with limited evidence suggesting *Rspo3* is downregulated in highly adipogenic MME^+^ FAPs (Fitzgerald et al., 2023).

In this study, we demonstrate that *Rspo* genes, particularly *Rspo2* and *Rspo3*, are expressed in murine FAPs and negatively regulate their adipogenic differentiation in an autocrine manner. *In vitro*, RSPO2 and RSPO3 act synergistically with canonical WNT3A to suppress FAP adipogenesis via β-catenin-dependent signaling. Consistently, *in vivo* intramuscular delivery of recombinant RSPO3 and WNT3A proteins in a glycerol induced muscle injury model reduced ectopic adipogenesis. Transcriptome analysis further supported these findings by showing suppression of adipogenesis-related transcriptional programs, including reduced expression of adipocyte-associated genes. In conclusion, these results identify a previously underappreciated role for RSPO2 and RSPO3 in regulating FAP adipogenesis through activation of canonical WNT signaling and suggest a potential therapeutic strategy to modulate FAP behavior, preserving their native functions in muscle homeostasis and regeneration while limiting pathological adipogenesis.

## 2 Materials and methods

### 2.1 Mice

Male mice (commercially obtained from ORIENT Bio, South Korea) were used throughout the study, with 6 to 10-week-old ICR mice for primary cells isolation were and 14 to 16-week-old C57BL/6J mice for single-cell RNA sequencing and *in vivo* experiments. For *Rspo3* conditional gene ablation, *Rspo3* ^*(f/f)*^ *;CAGG-Cre-ER* ^*TM*^ compound mice generated in-house by crossing *Rspo3* ^*(f/f)*^ mice (kindly provided by Dr. Christof Niehrs) (Kazanskaya et al., 2008) with *CAGGCre-ER* ^*TM*^ (obtained from The Jackson Laboratory, United States) mice (Hayashi and McMahon, 2002). For FAP-specific β-catenin gain of function experiments *in vitro*, *Pdgfra* ^*tm1.1(EGFP/cre/ERT2)Hyma/+*^ *;Ctnnb1* ^*tm1Mmt/+*^ mice generated in-house by crossing *Pdgfra* ^*tm1.1(EGFP/cre/ERT2)Hyma/+*^ (obtained from RIKEN BioResource Research Center, Japan) (Miwa and Era, 2015) with *Ctnnb1* ^*tm1Mmt/+*^ (kindly provided by Dr. Makoto Taketo) (Harada et al., 1999). Cre-mediated excision of exon 3 of the *Ctnnb1* gene containing phosphorylation sites results in stabilization of β-catenin.

All mouse experiments were conducted in accordance with protocols approved by the Institutional Animal Care and Use Committee (IACUC) of Soonchunhyang University. Mice were housed in a specific pathogen-free facility at the Soonchunhyang Institute of Medi-bio Science under a 12-h light/dark cycle and provided a standard chow diet.

### 2.2 Acute muscle injury

Glycerol (50% v/v, 50 μL, Sigma-Aldrich, St. Louis, MO, United States) was administered intramuscularly into tibialis anterior (TA) muscle to induce acute injury resulting in ectopic adipogenesis. Recombinant RSPO3 (10 μg, PeproTech, Rocky Hill, NJ, United States) and WNT3A (2 μg, PeproTech) proteins in 50 μL of phosphate-buffered saline (PBS) (Corning, Corning, NY, United States) for two consecutive days. PBS was injected as control. Tamoxifen (Sigma-Aldrich) in corn oil (20 mg/mL stock concentration) was administered intraperitoneally once daily for 5 consecutive days at a dose of 75 mg per kg of mouse body weight; corn oil was used as the control.

### 2.3 *In vivo* electroporation

siRNA electroporation was performed as previously described with minor modifications (Stephan et al., 2022). Briefly, mice were anesthetized, and 2 h prior to electroporation, TA muscles were injected with 15 μL of hyaluronidase solution (0.4 unit/μL, Sigma-Aldrich). Subsequently, 10 μg of siRNA targeting *Rspo2* or a non-targeting control were injected longitudinally into the TA muscle. Electroporation was then carried out by placing stainless-steel electrode needles (4 mm apart) parallel to the muscle axis and delivering eight square-wave pulses (80 V, 20 m/pulse) at 1 pulse/second using an ECM 830 Electroporation System (BTX, Holliston, MA, United States). Mice were allowed to recover on a heating pad after the procedure.

### 2.4 Isolation and culture of mouse FAPs and MuSCs

FAPs and MuSCs were isolated from whole hindlimb muscles of 6-week-old male ICR mice using previously described methods with minor modifications (Liu et al., 2015; Judson et al., 2017; Low et al., 2017). Briefly, the whole hindlimb muscles were harvested and processed by mechanical dissociation and enzymatic digestion with collagenase type II (Worthington Biochemical Corporation, Lakewood, NJ, United States) and dispase II (Roche Diagnostics, Mannheim, Germany). The resulting cell suspension was treated with red blood cell (RBC) lysis buffer (Sigma-Aldrich) and subjected to magnetic-activated cell sorting (MACS). Endothelial and hematopoietic cells were depleted by negative selection using CD31 and CD45 magnetic beads (Miltenyi Biotec, Bergisch Gladbach, Germany), respectively. FAPs and MuSCs were positively selected using SCA-1 and integrin alpha 7 magnetic beads (Miltenyi Biotec), respectively. Routinely, two to three mice were used to prepare primary cells sufficient for each experiment.

Mouse FAPs were cultured in growth medium consisting of high glucose Dulbecco’s Modified Eagle Medium (DMEM) (Corning) supplemented with 10% horse serum (HS) (Gibco, Grand Island, NY, United States), 20% fetal bovine serum (FBS) (Corning), 5 ng/mL basic fibroblast growth factor (bFGF) (PeproTech), and 1% penicillin-streptomycin (Corning) (Wosczyna et al., 2021). To induce CRE recombination, FAPs were treated with 10 μM of 4-hydroxytamoxifen (4-OHT) (Sigma-Aldrich) for 24 h.

For adipogenic differentiation, cells were first incubated in adipogenic induction medium (AIM) containing 0.5 mM 3-isobutyl-1-methylxanthine (IBMX) (Sigma-Aldrich), 0.25 μM dexamethasone (Sigma-Aldrich), and 10 μg/mL insulin (PeproTech) for 3 days, followed by culture in adipogenic maintenance medium (AMM) containing 10 μg/mL insulin for up to 10 days, with media changed every other day. Recombinant murine WNT3A (PeproTech), human R-spondin-2 (PeproTech), and R-spondin-3 proteins (PeproTech), which effectively enhance WNT signaling in murine cell systems, were applied during the first 3 days of differentiation at concentrations specified in the figure legends. The concentration and treatment duration of the recombinant proteins were selected to evaluate RSPO/WNT potentiation and to specifically target the early phase of adipogenic differentiation, respectively.

For fibrogenic differentiation, FAPs were maintained in growth medium for 2 days before being allowed to undergo spontaneous fibrogenesis in DMEM containing 10% FBS (Corning) and 1% penicillin-streptomycin (Corning) for 3 days (Liu et al., 2023) in the absence or presence of WNT and RSPO proteins.

### 2.5 Oil Red O staining

Oil Red O (ORO) staining was performed following a previously published protocol with minor modifications (Kraus et al., 2016). Briefly, FAP-derived adipocytes were washed with PBS, then fixed in 3.7% formaldehyde (Junsei Chemical Co., Ltd., Tokyo, Japan), stained with 0.5% (w/v) ORO (Sigma-Aldrich) solution, followed by hematoxylin (Sigma-Aldrich) counterstaining to visualize nuclei. Brightfield images were acquired at ×10 magnification using an ECLIPSE Ti-U inverted microscope (Nikon, Tokyo, Japan). For quantitative analysis, the retained ORO dye was extracted using 100% isopropanol (Carlo Erba Reagents, Cornaredo, Italy), and absorbance was measured at 490 nm using a Multiskan GO microplate spectrophotometer (Thermo Fisher Scientific, Waltham, MA, United States).

### 2.6 Western blot analysis

Cultured cells were either lysed directly in Laemmli buffer and denatured at 95 °C for 5 min or, for time-course experiments, lysed in RIPA buffer supplemented with protease and phosphatase inhibitors prior to denaturation. Proteins were separated by SDS-PAGE, transferred to PVDF membranes (Cytiva, Marlborough, MA, United States), and blocked with 5% skim milk (BD Difco, Sparks, MD, United States) before overnight incubation with primary antibodies diluted in 1% bovine serum albumin (BSA) (BioShop, Burlington, ON, Canada) at 4 °C. Membranes were then incubated with HRP-conjugated secondary antibodies, and signals were detected using an enhanced chemiluminescence Amersham™ ECL™ Prime Western blot detection reagent (Cytiva) and imaged digitally with the Amersham Imager 600 (GE HealthCare, Chicago, IL, United States) at the Soonchunhyang Biomedical Science Core Facility. Primary antibodies used included anti-PPARɣ (1:1,000 dilution, Cell Signaling Technology/CST, Danvers, MA, United States), C/EBPα (1:1,000 dilution, CST), FABP4 (1:1,000 dilution, CST), β-catenin (1:1,000 dilution, CST), β-actin (1:8,000 dilution, Sigma-Aldrich), vinculin (1:1,000 dilution, CST), COL1A1 (1:1,000 dilution, CST), CTGF (1:500, Santa Cruz Biotechnology, Inc., Dallas, TX, United States).

Densitometric analysis of Western blot densitometric was performed using Fiji/ImageJ. For each independent immunoblot, the intensity of each target protein band was normalized to the corresponding loading control, and the resulting values were expressed relative to the corresponding control from the same immunoblot, which was assigned a value of 1.00. Relative protein values are displayed below the respective lanes. Due to the small sample size (n = 2), densitometric quantification is presented descriptively without being subjected to inferential statistical analysis.

### 2.7 siRNA transfection

siRNA targeting mouse *Rspo2*, *Rspo3*, and *Ctnnb1*, along with non-targeting control, were obtained commercially from Dharmacon (Lafayette, CO, United States). The siRNA targeting each gene is a pool of four independent siRNAs specific to that gene. The sequences of the individual siRNA are provided in the Supplementary Material (see Supplementary Table S2). Fifteen pmol of siRNA targeting *Rspo2*, *Rspo3* and *Ctnnb1* were used to achieve effective gene knockdown. Reverse transfection was performed using Lipofectamine™ RNAiMAX (Invitrogen, Carlsbad, CA, United States) complexed with siRNAs diluted in serum-free Opti-MEM^TM^ medium (Gibco) at a 1:3 ratio. FAPs were seeded in growth medium, siRNA-lipofectamine complexes were added immediately to initiate transfection. After 18–20 h of incubation at 37 °C in 5% CO_2_ incubator, the medium was replaced with fresh growth medium to allow recovery, and adipogenic differentiation was induced.

### 2.8 RNA isolation and quantitative RT-PCR

Total RNA for droplet digital polymerase chain reaction (ddPCR) was isolated from freshly isolated FAPs and SCs using the RNAqueous^TM^-Micro Total RNA Isolation Kit (Thermo Fisher Scientific) while RNA for RT-qPCR was extracted using TRIzol™ reagent (Ambion, Austin, TX, United States). cDNA was synthesized using the ProtoScript® First Strand cDNA Synthesis Kit (New England Biolabs, Ipswich, MA, United States).

ddPCR was performed using the QX200 system (Bio-Rad Laboratories, Hercules, CA, United States) with EvaGreen Supermix (Bio-Rad Laboratories). Droplets containing gene-specific primers and DNA template (5 ng in 22 μL) were generated using the QX200 droplet generator. After PCR amplification, positive droplets were identified using QX200 droplet reader and the data were analyzed on QuantaSoft version 1.7.4 (Bio-Rad Laboratories). RT-qPCR was conducted using PowerUp™ SYBR™ Green Master Mix (Thermo Fisher Scientific) on a StepOnePlusTM Real-Time PCR System (Thermo Fisher Scientific). All primers used for ddPCR and RT-qPCR were commercially synthesized by Cosmogenetech (Seoul, South Korea). Their sequences are provided in Supplementary Material (see Supplementary Table S1).

### 2.9 Tissue processing and histology

TA muscles were fixed in 4% paraformaldehyde (Sigma-Aldrich) for up to 20 h and washed in distilled water. Samples were then dehydrated through a graded ethanol series (Daejung Chemicals and Metals Co., Ltd., Siheung, Korea), cleared in xylene (Daejung Chemicals and Metals Co.), embedded in paraffin (Paraplast) (Leica Biosystems, Nussloch, Germany), and sectioned at 6 μm thickness onto glass slides. Prior to staining, sections were deparaffinized and rehydrated.

Rehydrated sections were stained with hematoxylin for 1 min, rinsed in tap water, and blued with 0.2% ammonium hydroxide (Sigma-Aldrich), followed by an additional rinse. Sections were then immersed in 70% ethanol for 30 s, counterstained with eosin (BBC Biochemical, Mount Vernon, WA, United States) for 1 min, dehydrated through graded ethanol, cleared with HistoChoice® Clearing Agent (Sigma-Aldrich), and mounted using VectaMount Permanent Mounting Medium (Vector Laboratories, Newark, CA, United States). Tissue images were acquired using a Nikon ECLIPSE Ti-U inverted microscope.

### 2.10 Immunofluorescence staining

Rehydrated sections were subjected to antigen retrieval, followed by blocking with 5% serum in PBS containing 0.2% Triton X-100 (Sigma-Aldrich) for 1 h. Sections were then incubated overnight with anti-FABP4 primary antibody (1:50 dilution, Abcam, Cambridge, United Kingdom) or anti-PLIN1 primary antibody (1:80 dilution, CST), followed by incubation with Cy3-conjugated donkey anti-rabbit antibody (1:750 dilution, Jackson ImmunoResearch, West Grove, PA, United States) for FABP4 or Alexa Fluor 488-conjugated donkey anti-rabbit antibody (1:750, Jackson ImmunoResearch) for PLIN1. Sections were then mounted with DAPI-containing Fluoroshield™ medium (Sigma-Aldrich). Imaging was performed under identical exposure and gain settings across all groups.

For immunofluorescence staining of FAPs, cultured FAPs were fixed 3.7% formaldehyde (Junsei Chemical Co., Ltd.), permeabilized with 1% IGEPAL (Sigma-Aldrich)/PBS, and blocked for 30 min at room temperature with 5% donkey serum and 3% BSA in PBS. Cells were then incubated overnight with an anti-COL1A1 antibody (1:200, CST), followed by incubation with Cy3-conjugated donkey anti-rabbit antibody (1:750, Jackson ImmunoResearch) and counterstaining with DAPI.

### 2.11 Single-cell RNA sequencing (scRNAseq)

Single cells were isolated from whole hind limb muscle of 9-week-old C57BL/6J mice and captured using the 10x Chromium Single Cell Controller (10x Genomics, Pleasanton, CA, United States), targeting 10,000 cells per mouse. scRNA-seq cDNA libraries were generated using NextGEM Single Cell 3′ Library and Gel bead kit v3.1 following manufacturer’s protocol and sequenced on a NovaSeq 6,000 platform (Illumina, San Diego, CA, United States) at a depth of ∼500 million reads per library. Raw sequencing data were processed using the Cell Ranger pipeline (10x Genomics) for alignment to the mouse reference genome (mm10; GENCODE vM23/Ensembl 98) and generation of gene-cell count matrices. Processed data were analyzed in Loupe Browser (10x Genomics) for visualization of cell clusters and marker gene expression. Cells were grouped into ten clusters using k-means clustering (k = 10) and annotated based on canonical markers, including *Pdgfra* for FAPs and *Pax7* for MuSCs.

### 2.12 Transcriptome analysis

Total RNAs were isolated from the glycerol-injured TA muscles injected with RSPO3/WNT3A and control vehicle using TRIzol reagent (Ambion). RNA sequencing and identification of differentially expressed genes (DEGs) were carried out by BGI Genomics (Shenzhen, China) using DESeq2 software (Love et al., 2014). We selected DEGs filtering with the criteria; *q*-value (FDR) ≤ 0.05 and log_2_FC ≥ 0.58 (≈1.5-fold or more). Pathway enrichment analyses, including WikiPathways, Reactome, TRRUST transcription factors, KEGG and Gene ontology, were conducted using the ENRICHR online tool (https://maayanlab.cloud/Enrichr/). Data were visualized using R packages (RStudio; ggplot2 and Enhanced Volcano) and GraphPad Prism v9.0 (GraphPad Software).

### 2.13 Image quantification and statistical analysis

For immunofluorescence images quantitative analysis, multiple non-overlapping fields per section were analyzed using in Fiji/ImageJ. Individual FABP4^+^ cells were manually outlined to obtain region of interests (ROI), mean intensity, and integrated density. Background fluorescence was determined from signal-free regions and used to calculate corrected total cell fluorescence (CTCF) as: Integrated Density - (ROI area x mean background intensity) (Gavet and Pines, 2010; Viloria et al., 2020; Wang et al., 2024; Emendato et al., 2025). CTCF values were stratified using pooled P10/P90 thresholds into low (<P10), medium (P10-P90), and high (>P90) categories. The percentage of cells in each category was calculated per mouse and compared between control PBS- and RSPO3/WNT3A-injected groups.

PLIN1-positive adipocytes were identified by the presence of a distinct PLIN1-positive rim surrounding lipid-droplets. The injured area of each section was determined from the corresponding H&E-stained muscle section. PLIN-positive adipocytes were counted within the injured regions and normalized to the corresponding total injured area. Data are expressed as PLIN1-positive adipocyte density (cells/mm^2^ of injured area).

All data are presented as mean ± standard error of the mean (SEM) unless otherwise indicated and analyzed using GraphPad Prism v9.0 (GraphPad Software, Boston, MA, United States). Comparisons between two groups were analyzed using unpaired two-tailed Student’s t-test, while multiple-group comparisons were analyzed by one-way ANOVA followed by Tukey’s *post hoc* test. Statistical significance was defined using *p* or *q* and denoted as follows: * ≤0.05; ** ≤0.01; *** ≤0.001; **** ≤0.0001.

For the *in vivo* studies, a retrospective power analysis was performed using mouse-level mean CTCF values from the first *in vivo* experiment involving RSPO3/WNT3A administration to glycerol-injured TA muscle. Cohen’s d was calculated as the difference between group means divided by the pooled standard deviation. With four mice per group, a two-sided α of 0.05, and the observed Cohen’s d, the calculated statistical power was approximately 99.97%. Normality was assessed using the Shapiro-Wilk test for *in vivo* experiments analyzed using parametric tests.

## 3 Results

### 3.1 Expression of the *Rspo* gene family in FAPs

To initiate our investigation on RSPO function in FAPs, we first determined expression of the *Rspo* gene family in muscle-resident cells, including FAPs, using single-cell RNA sequencing (scRNA-seq). Among the ten identified cell clusters (Figure 1A), *Rspo* gene expression - at varying level - was primarily observed in FAPs (orange-colored cell cluster demarcated by the dotted black line), as indicated by the expression of *Pdgfra, Thy1* and *Ly6a*, but not in myofibroblasts (green-colored cell cluster demarcated by the dotted purple line) (Figures 1B,C). Notably, *Rspo3* showed the highest expression among all *Rspo* genes in FAPs, whereas *Rspo4* expression was not detected.

**FIGURE 1:**
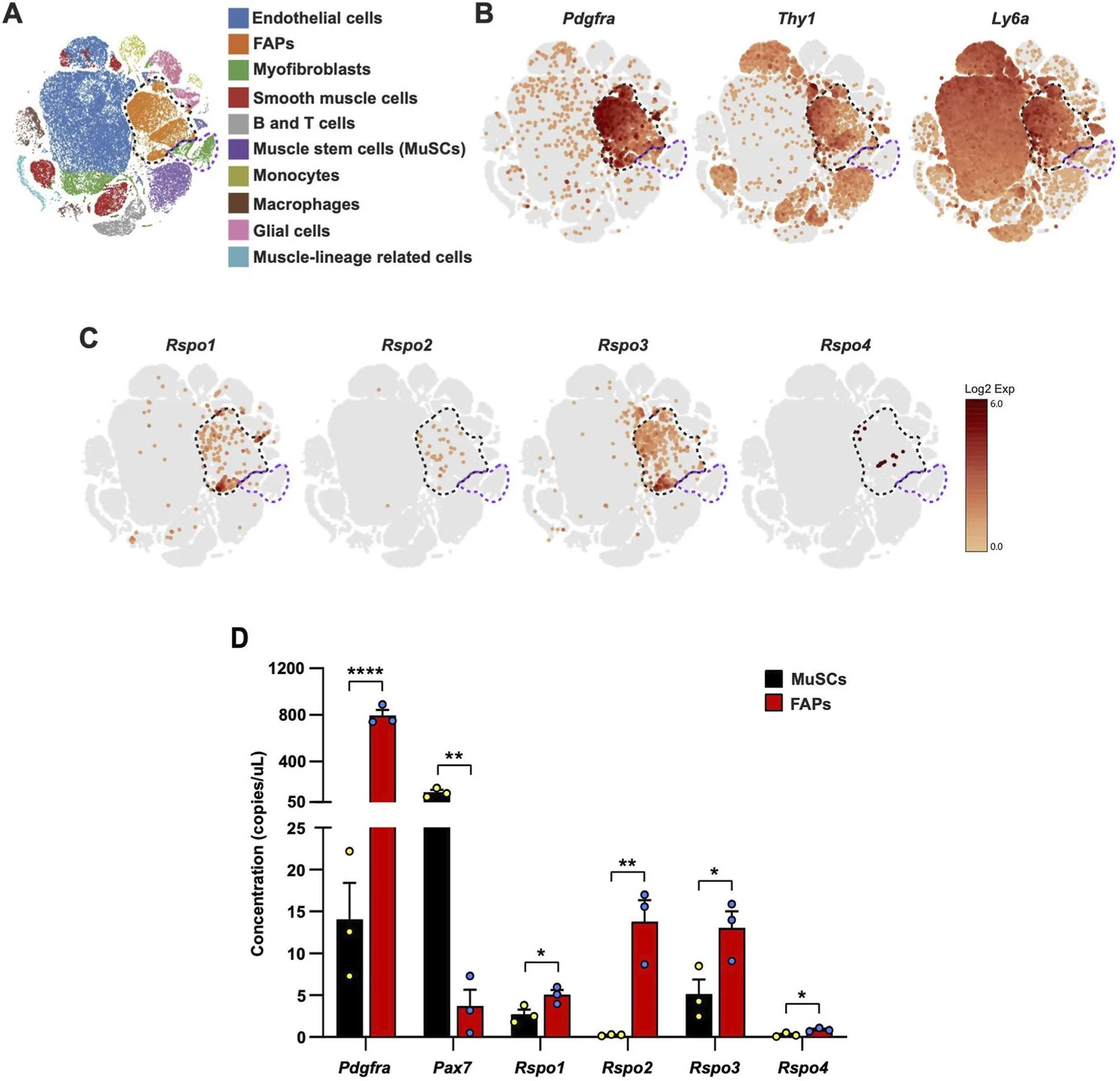
Integrated transcriptional profiling reveals enriched expression of *Rspo* gene family members in FAPs. Single-cell RNA sequencing data from uninjured whole-leg muscle isolated from normal C57BL/6J mice (n = 6, 70,279 cells in total) are shown. **(A)** t-SNE projection of 10 identified cell types, with each color representing an annotated cell cluster. The dotted black and purple lines demarcate the FAP and myofibroblast populations, respectively. **(B)** t-SNE plots showing *Pdgfra*, *Thy1*, *Ly6a* (Sca-1) expression. FAPs are positive for *Pdgfra, Thy1* and *Ly6a* expression, whereas myofibroblast-like cells within *Pdgfra*-positive population are negative for *Thy1* expression. **(C)** Expression of *Rspo* gene family members in FAP population, excluding myofibroblast-like cells. Color intensity scale bar represents gene expression level in log_2_ scale. **(D)** Expression of the *Rspo* family member in isolated FAPs by ddPCR. MuSCs were used to compare *Rspo* expressions. Both *Pdgfra* and *Pax7* expression was confirmed for FAPs and MuSCs, respectively. RNA samples from FAPs and MuSCs were prepared from three independent biological replicates (n = 3). The ddPCR data are presented as mean ± SEM of absolute concentration (copies/uL) and individual dots represent each biological replicate. Statistical significance was determined using an unpaired two-tailed Student’s t-test. *p ≤ 0.05; **p ≤ 0.01; ***p ≤ 0.001; ****p ≤ 0.0001.

To further validate the scRNA-seq results, we isolated FAPs from whole leg muscles, along with MuSCs, and analyzed *Rspo* gene expression by ddPCR (Figure 1D). Consistent with the scRNA seq findings, *Rspo3* expression was the most abundant. Interestingly, *Rspo2* expression level in isolated FAPs was nearly equivalent to that of *Rspo3*. In comparison, *Rspo* gene expression in MuSCs was relatively low, with weak expression of both *Rspo2* and *Rspo3*.

### 3.2 RSPO2 and RSPO3 inhibit adipogenic differentiation of FAPs

Because FAPs possess the capacity to differentiate into either fibroblasts or adipocytes, we first investigated whether RSPO2 and RSPO3 affects adipogenesis. As RSPO family members are known to potentiate canonical WNT signaling (Ruffner et al., 2012; Hao et al., 2016), we treated FAPs with RSPO2 or RSPO3 in the presence of WNT3A.

Adipogenic differentiation was induced by culturing isolated FAPs in adipogenic induction medium (AIM) for 3 days, followed by adipogenic maintenance medium (AM) for 7 days (Figure 2A). Recombinant RSPOs and WNT3A were added during the AIM phase. Cells were collected at multiple time points during differentiation.

**FIGURE 2:**
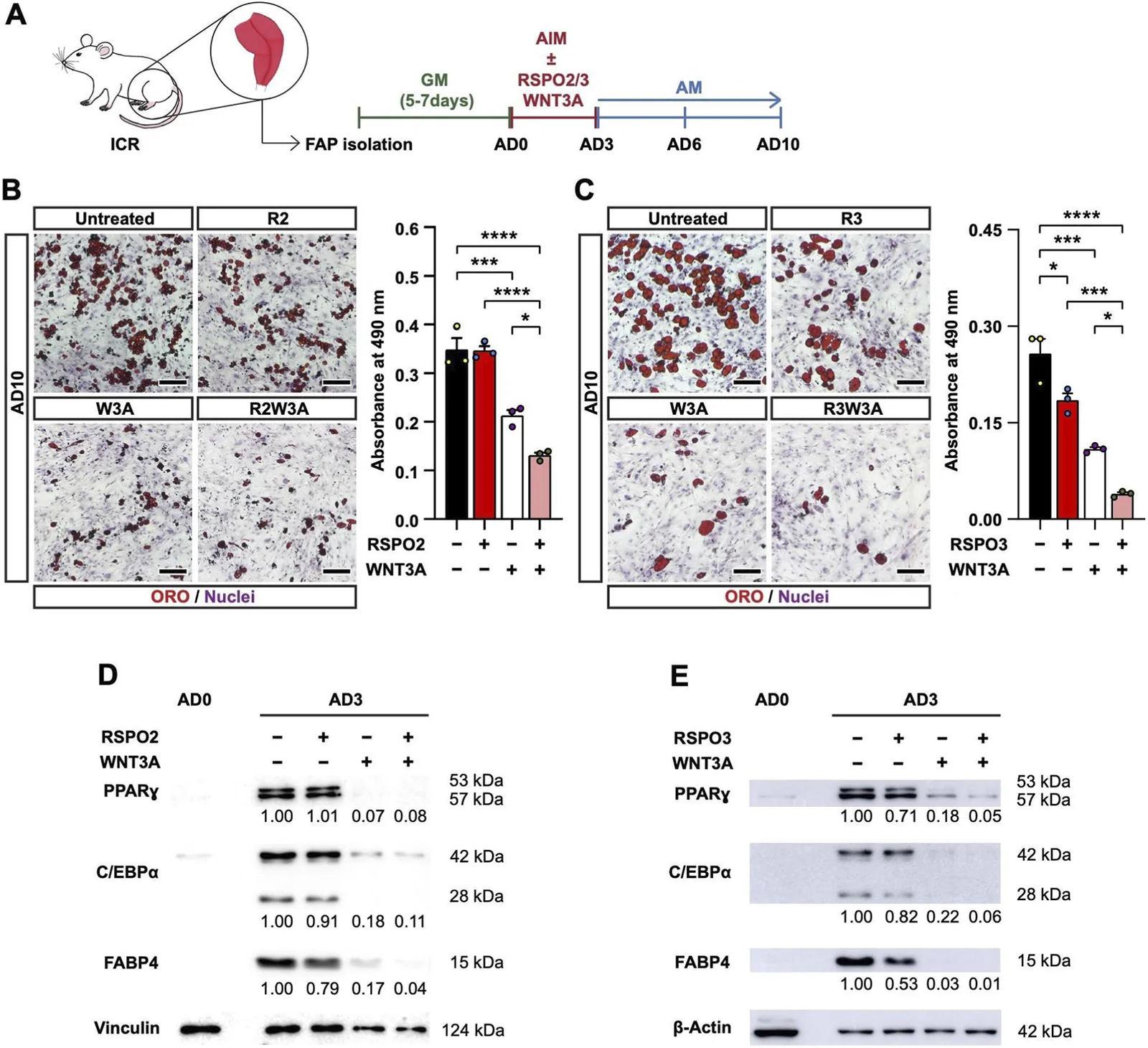
The inhibitory effect of RSPO2/WNT3A and RSPO3/WNT3A treatments on FAP adipogenesis. **(A)** Experimental design for investigating the effects of RSPO2/WNT3A and RSPO3/WNT3A treatments during FAP adipogenic differentiation. RSPO2 (50 ng/mL), RSPO3 (50 ng/mL), and WNT3A (10 ng/mL) were added to the adipogenesis inducing medium (AIM) for 3 days, followed by adipogenic differentiation medium (AM) up to 10 days (AD10). **(B,C)** Oil Red O (ORO) staining analysis at AD10 following RSPO2/WNT3A **(B)** or RSPO3/WNT3A **(C)** treatment, including representative images (scale bar = 200 μm) and corresponding absorbance quantification. Samples were prepared from three independent biological replicates (n = 3). ORO quantification data presented as mean ± SEM of absorbance values at 490 nm after baseline subtraction using AD0 values. **(D,E)** Western blot analysis of adipogenic marker expression for the same treatment groups: RSPO2/WNT3A **(D)** and RSPO3/WNT3A **(E)** at AD3. Densitometric quantification was performed using two independent biological replicates (n = 2). The resulting values are presented as numbers below the corresponding lanes, indicating the relative fold change compared with the control from the same immunoblot (control value set to 1.00), after normalization to Vinculin for RSPO2/WNT3A treatment and β-Actin for RSPO3/WNT3A treatment. Both Vinculin and β-Actin are reliable normalization markers and were used interchangeably across experiments. All bar graphs include individual dots representing each biological replicate. Statistical significance was determined using one-way ANOVA with Tukey’s *post hoc* test. *p ≤ 0.05; **p ≤ 0.01; ***p ≤ 0.001; ****p ≤ 0.0001.

Oil Red O (ORO) staining at days 7 (AD7) and 10 (AD10) was performed to assess lipid accumulation. While WNT3A alone effectively inhibits lipid accumulation, RSPO2 or RSPO3 alone had little or no effect at AD10 (Figures 2B,C). However, in the presence of WNT3A, both RSPO2 and RSPO3 prominently enhanced the inhibitory effect. At AD7, RSPO2 and RSPO3 showed similar effects on lipid accumulation (Supplementary Figures S1A, B).

To determine whether these effects of RSPO2 and RSPO3 on lipid accumulation occur during early differentiation stage, canonical adipogenesis markers were analyzed by Western blotting at AD3 and AD6. At AD3, expression of PPARɣ and C/EBPα, two master regulators of adipogenesis (Gerstner et al., 2024), was mildly reduced by RSPO2 or RSPO3 alone, but substantially suppressed by WNT3A alone or in combination with RSPO2 or RSPO3 (Figures 2D,E; Supplementary Figures S2A,B). FABP4, a marker for adipocytes (Shan et al., 2013), showed a similar pattern. Comparable results were observed at AD6 (Supplementary Figures S1C,D). Together, these results indicate that RSPO2 and RSPO3 potentiate WNT3A-mediated inhibition of adipogenesis in FAPs.

### 3.3 Reduced *Rspo2* and *Rspo3* expression enhances adipogenesis of FAPs

To investigate the role of endogenous *Rspo2* and *Rspo3*, we performed siRNA-mediated gene knockdown experiments (Figure 3A). Knockdown efficiency was confirmed by RT-qPCR, demonstrating specific and effective reduction of each *Rspo* gene (Figure 3B). Because RSPO2 and RSPO3 exhibited similar effects, we also performed simultaneous knockdown, achieving over 60% reduction of both genes.

**FIGURE 3:**
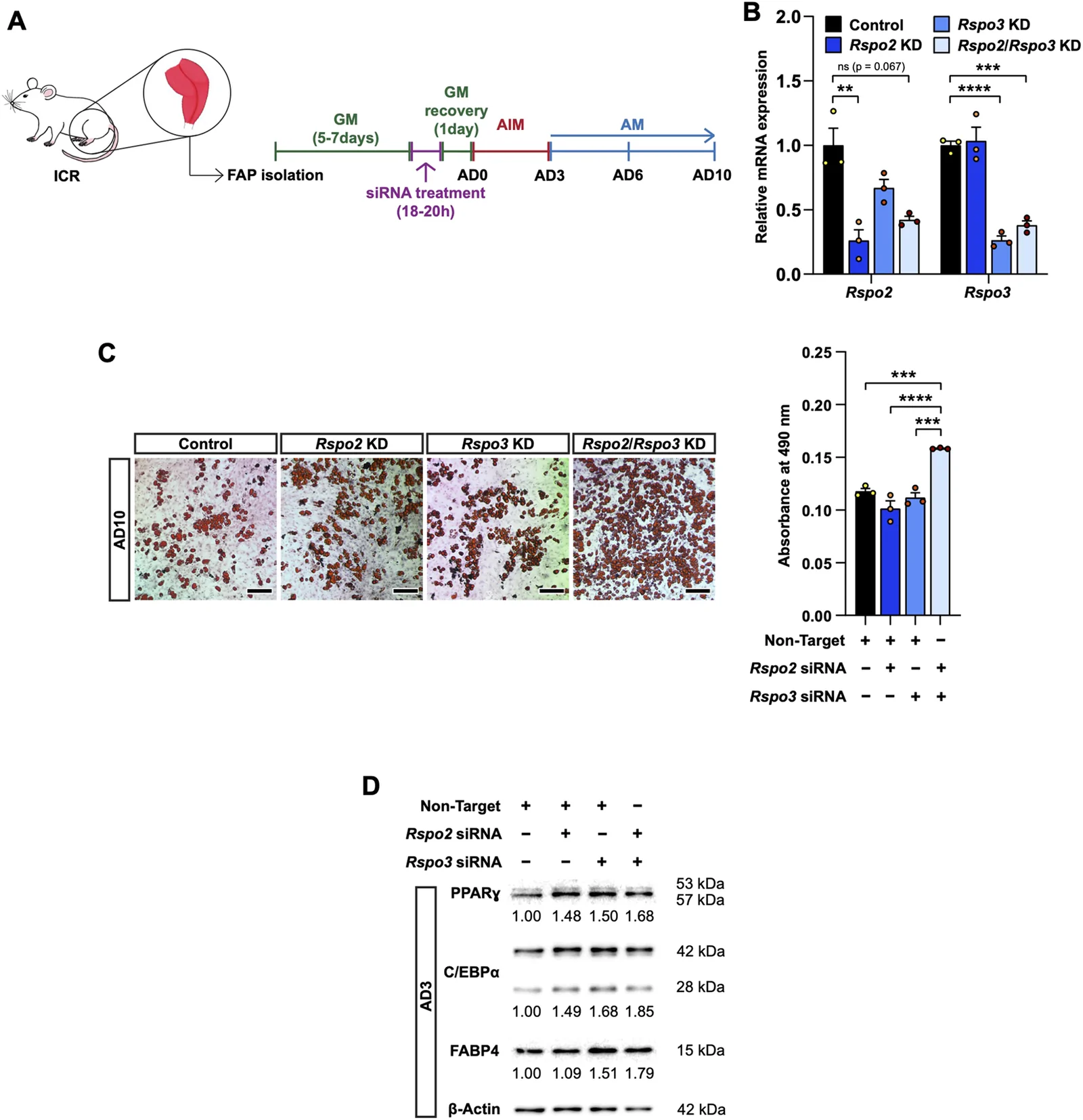
Endogenous *Rspo2* and *Rspo3* gene knockdown promotes FAP adipogenesis. **(A)** Experimental design for *Rspo2* and *Rspo3* siRNA knockdown in FAPs during adipogenic differentiation. Cultured FAPs were transfected with siRNA for 18–20 h, followed by recovery in growth medium for 1 day before initiating adipogenic differentiation. Adipogenic differentiation was induced with culturing FAPs in AIM for 3 days and subsequently AM for up to 7 days. **(B)** siRNA-mediated knockdown efficiency for *Rspo2* and *Rspo3* was assessed by RT-qPCR in four groups: non-target siRNA transfected as control, *Rspo2* siRNA-transfected (*Rspo2* KD), *Rspo3* siRNA-transfected (*Rspo3* KD), and *Rspo2/Rspo3* siRNA-transfected (*Rspo2*/*Rspo3* KD). RNA samples for each group were prepared from three independent biological replicates (n = 3). **(C)** Representative images (scale bar = 200 μm) and quantification of ORO staining analysis at AD10. Absorbances at 490 nm were measured from three independent biological replicates (n = 3) and are presented after baseline subtraction using AD0 values. **(D)** Western blot analysis of adipogenic marker expression in *Rspo2* and *Rspo3* knockdown cells at AD3. Corresponding densitometric quantification was performed using two independent biological replicates (n = 2). The resulting values are presented as numbers below the corresponding lanes, indicating the relative fold change compared with the control from the same immunoblot (control value set to 1.00), after normalization to β-Actin. All bar graphs include individual dots representing each biological replicate. Statistical significance was determined using one-way ANOVA with Tukey’s *post hoc* test. *p ≤ 0.05; **p ≤ 0.01; ***p ≤ 0.001; ****p ≤ 0.0001.

At AD10, FAPs transfected with non-targeting, *Rspo2*-specific, or *Rspo3*-specific siRNA showed comparable lipid accumulation (Figure 3C). However, dual knockdown of *Rspo2* and *Rspo3* highly increased lipid accumulation. Notably, at AD7, individual knockdown of *Rspo2* or *Rspo3* also increased lipid accumulation compared to non-targeting control (Supplementary Figure S3A).

Western blot analysis showed that reduced expression of *Rspo2*, *Rspo3* or both led to increased expression of adipogenic markers at AD3 (Figure 3D; Supplementary Figure S2C) and AD6 (Supplementary Figure S3B). These findings suggest that endogenous *Rspo2* and *Rspo3* play redundant, anti-adipogenic roles, likely through an autocrine mechanism.

### 3.4 RSPO3/WNT3A-mediated inhibition of adipogenesis is β-catenin-dependent

To determine whether RSPO-mediated inhibition of adipogenesis depends on canonical WNT signaling, we examined adipogenic differentiation in FAPs with reduced β-catenin level (Figure 4A). FAPs were transfected with siRNA targeting *Ctnnb1* (encoding β-catenin) and induced to differentiate in the presence of RSPO3 and/or WNT3A. β-catenin knockdown efficiency (60%–80%) was confirmed by Western blot (Figure 4C; Supplementary Figures S2D, S4C). Reduction of β-catenin partially reversed the inhibitory effects of RSPO3/WNT3A on lipid accumulation at AD10 (Figure 4B). Similarly, suppression of adipogenic marker expression was partially restored at AD3 (Figure 4C; Supplementary Figure S2D). Comparable results were observed at AD6 (Supplementary Figure S4C).

**FIGURE 4:**
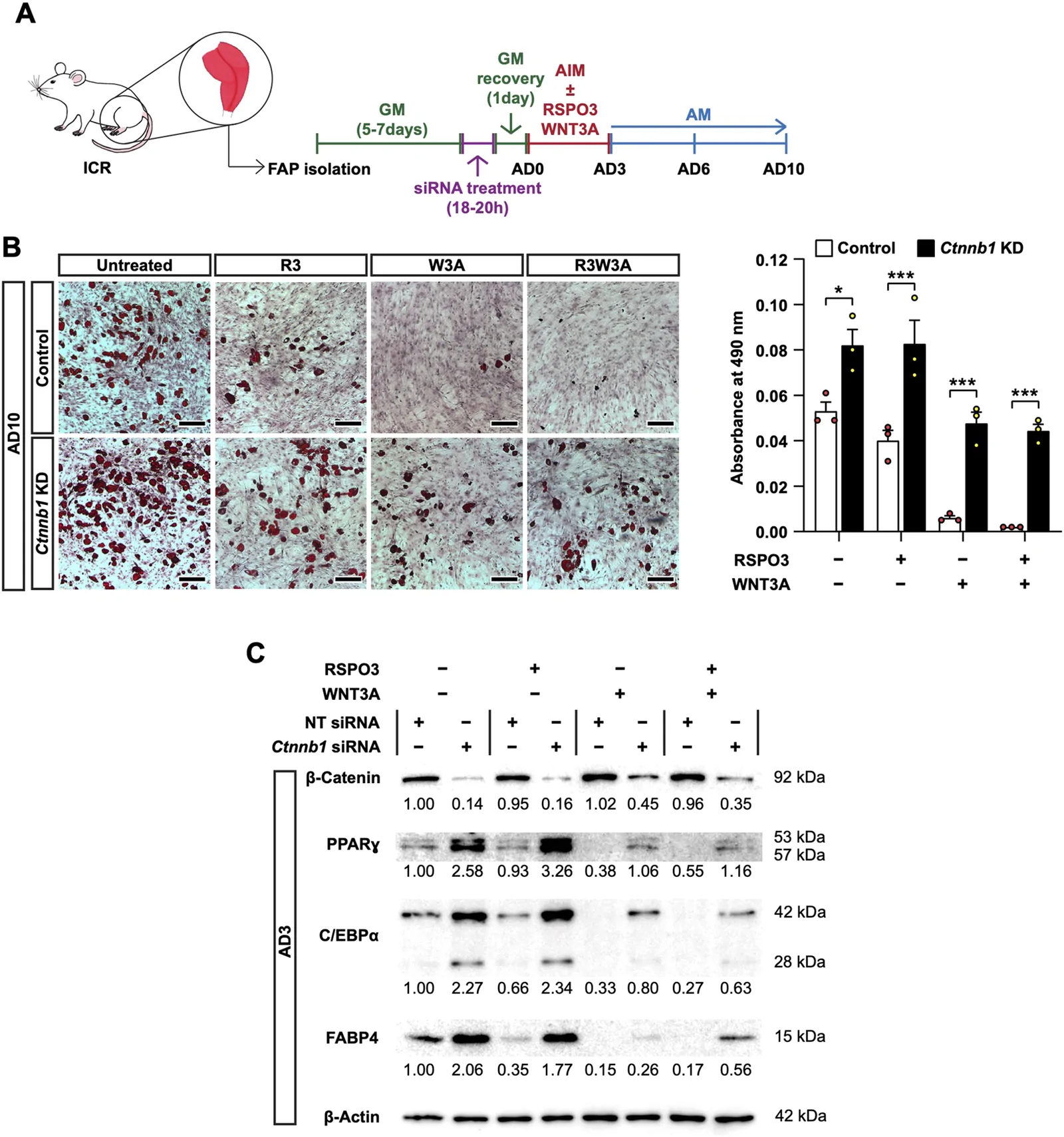
The inhibitory effect of RSPO3/WNT3A on FAP adipogenesis is mediated through canonical WNT signaling in a β-catenin-dependent manner. **(A)** Experimental design for *Ctnnb1* knockdown in FAPs. The *Ctnnb1* siRNA was transfected into FAPs in growth medium. After 1 day of recovery, FAPs were induced for adipogenic differentiation by culturing in AIM for 3 days followed by AM for up to 7 days. RSPO3 (50 ng/mL) and WNT3A (10 ng/mL) were added in AIM for 3 days. **(B)** Representative images (scale bar = 200 μm) and quantification of ORO staining analysis at AD10. Absorbances at 490 nm were measured from three independent biological replicates (n = 3) and are presented after baseline subtraction using AD0 values. **(C)** Western blot analysis showing β-catenin knockdown efficiency and expression of adipogenic markers in *Ctnnb1* knockdown FAPs following RSPO3/WNT3A treatment at AD3. Corresponding densitometric quantification was performed using two independent biological replicates (n = 2). The resulting values are presented as numbers below the corresponding lanes, indicating the relative fold change compared with the control from the immunoblot (control value set to 1.00), after normalization to Vinculin. All bar graphs include individual dots representing each biological replicate. Statistical significance was determined using one-way ANOVA with Tukey’s *post hoc* test. *p ≤ 0.05; **p ≤ 0.01; ***p ≤ 0.001; ****p ≤ 0.0001.

We next examined FAPs expressing a constitutively active β-catenin (Harada et al., 1999). These cells were derived from mice carrying a β-catenin gain of function allele (exon3 deletion) under control of a tamoxifen-inducible *Pdgfra-CreERT* system. Activation was induced by 4-hydroxytamoxifen (4-OHT) prior to differentiation (Figure 5A). β-catenin activation was confirmed by increased expression of *Axin2*, a known downstream target (Huraskin et al., 2016), showing *∼*60-fold and ∼30-fold at AD3 and AD10, respectively (Figure 5B).

**FIGURE 5:**
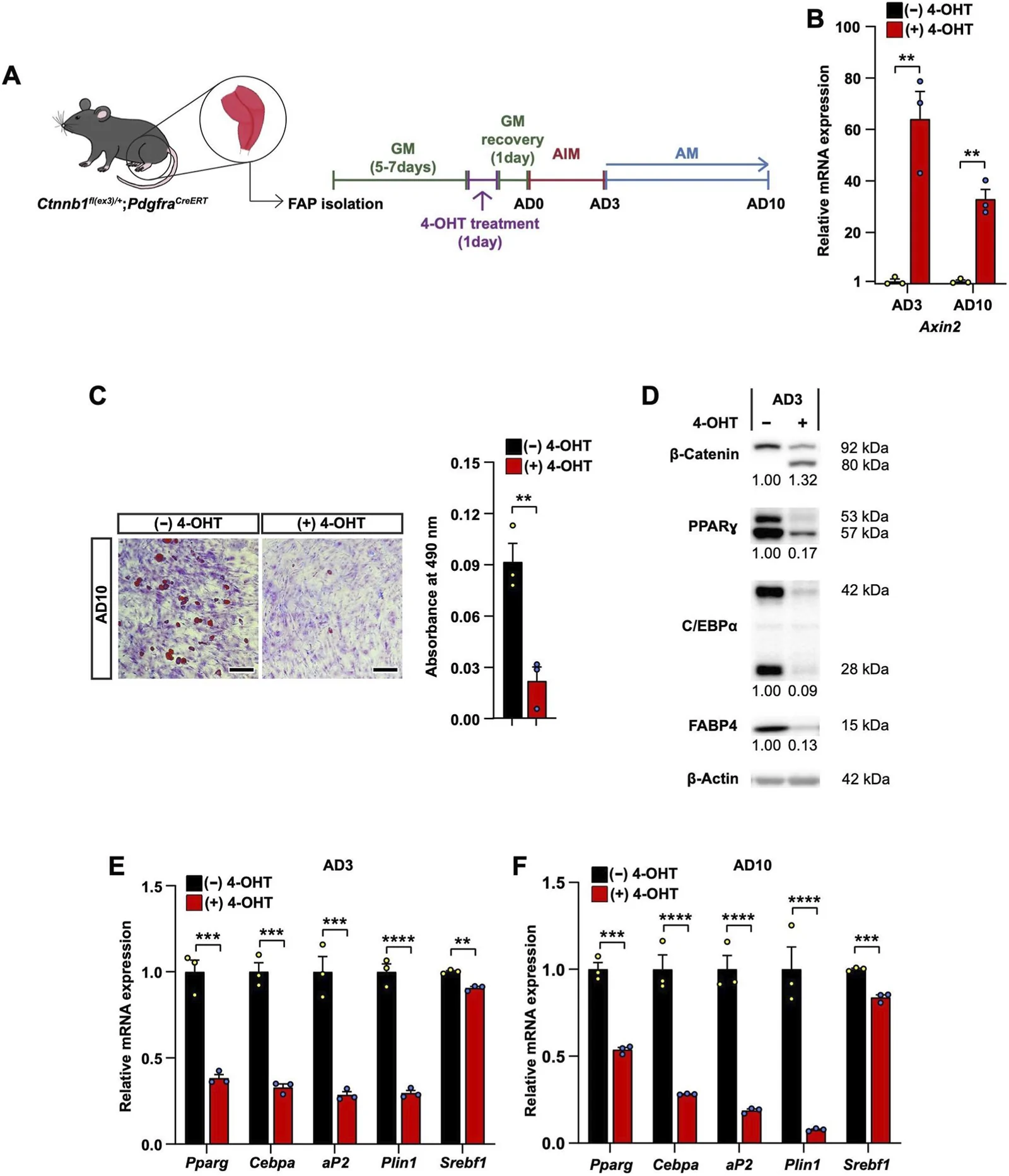
Expression of a constitutively active form of β-catenin suppresses adipogenic differentiation in FAPs. **(A)** Experimental design for producing a constitutively active form of β-catenin in FAPs during adipogenic differentiation. FAPs were isolated from *Ctnnb1* ^*tm1Mmt/+*^ *;Pdgfra* ^*tm1.1(EGFP/cre/ERT2)Hyma/+*^ (*Ctnnb1* ^*fl(ex3)/+*^ *;Pdgfra* ^*CreERT*^) compound mice. Cultured FAPs were treated with 4-OHT (10 μM) diluted in growth medium for 24 h to induce the Cre-dependent recombination. FAPs without 4-OHT treatment were used as control group. FAPs were cultured in AIM for 3 days and subsequently in AM for up to 7 days before harvesting cells. **(B)** RT-qPCR analysis of expression of β-catenin target gene, *Axin2*, in 4-OHT treated FAPs. Expression level was measured using three independent biological replicates (n = 3) and is normalized to *Actab*. **(C)** Representative images (scale bar = 200 μm) and quantification of ORO staining analysis at AD10. Absorbances at 490 nm were measured using three independent biological replicates (n = 3) and are presented after baseline subtraction using AD0 values. **(D)** Western blot analysis of β-catenin and adipogenic marker expression in 4-OHT treated FAPs at AD3. Corresponding densitometric quantification was performed using two independent biological replicates (n = 2). The resulting values are presented as numbers below the corresponding lanes, indicating the relative fold change compared with the control from the same immunoblot (control value set to 1.00), after normalization to β-Actin. **(E,F)** RT-qPCR analysis of adipogenic gene expression at AD3 **(E)** and AD10 **(F)**. Expression levels were measured using three independent biological replicates (n = 3) and are normalized to *Actab* and presented relative to the untreated control. All bar graphs include individual dots representing each biological replicate. Statistical significance was determined using one-way ANOVA with Tukey’s *post hoc* test. *p ≤ 0.05; **p ≤ 0.01; ***p ≤ 0.001; ****p ≤ 0.0001.

Under these condition, adipogenic differentiation was markedly suppressed. Lipid accumulation was substantially reduced at AD10 (Figure 5C), and adipogenic marker expression was strongly decreased at both the protein (Figure 5D; Supplementary Figure S2E) at AD3 and mRNA levels (Figures 5E,F) at both AD3 and AD10. These results demonstrate that activation of canonical WNT/β-catenin signaling is critical for RSPO3/WNT3A-mediated inhibition of adipogenesis.

### 3.5 RSPO3/WNT3A attenuates glycerol-induced ectopic adipogenesis *in vivo*

To assess the *in vivo* relevance of RSPO3/WNT3A, we used a glycerol-induced muscle injury model that promotes ectopic adipogenesis (Reggio et al., 2020). RSPO3 and WNT3A proteins were injected into the injured TA muscle (Figure 6A). Adipogenesis was assessed by FABP4 immunofluorescence, with Laminin staining marking the myofiber boundaries (Figure 6B). Quantification was performed using corrected total fluorescence (CTCF) of FABP4-positive cells.

**FIGURE 6:**
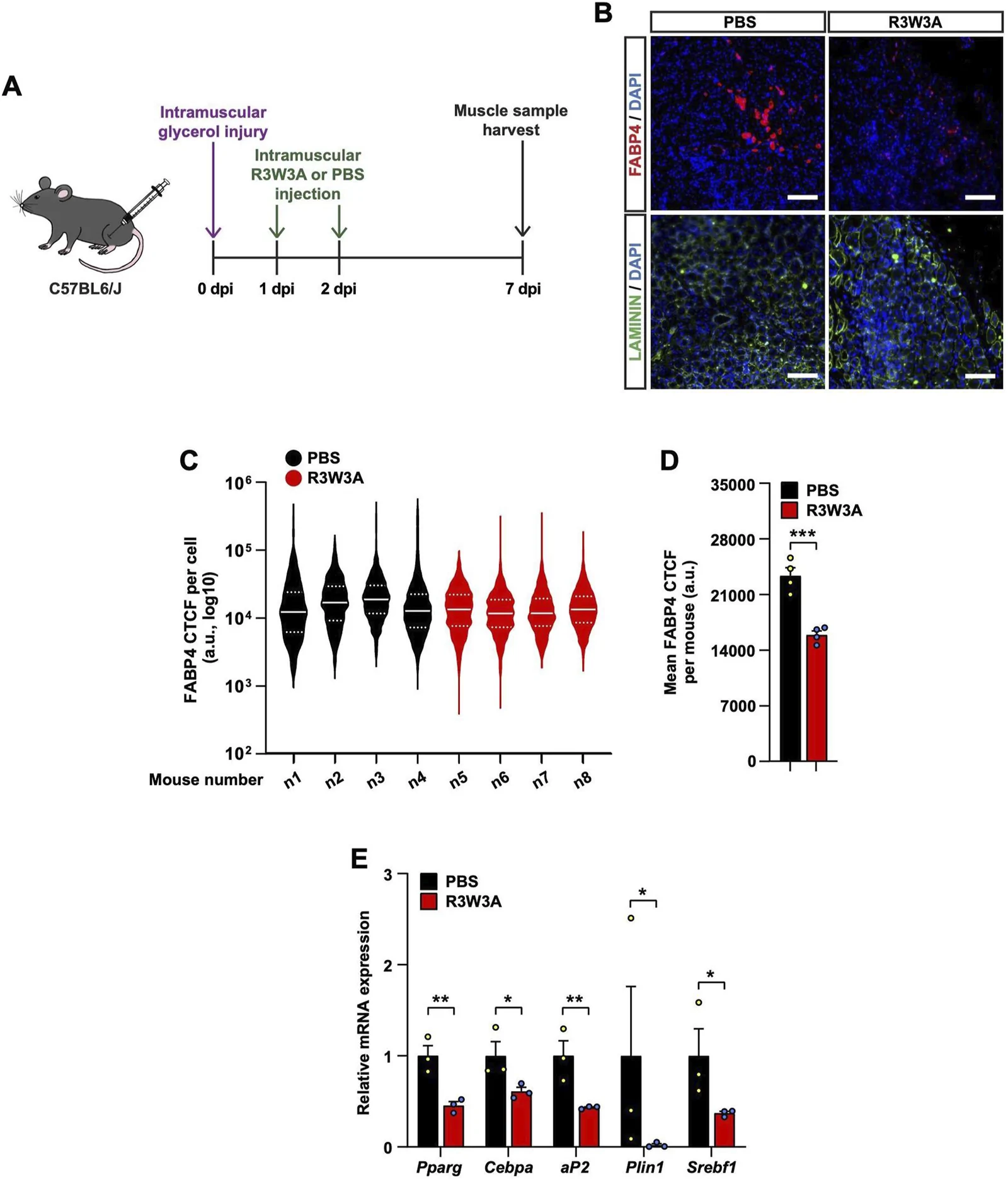
RSPO3/WNT3A reduces ectopic adipocyte accumulation induced by glycerol injury in TA muscle. **(A)** Experimental design for RSPO3/WNT3A administration in glycerol-injury mouse model. Glycerol was administered intramuscularly into TA muscle to induce acute injury. Twenty four hours later, RSPO3 (1 μg/μL, 10 μL) and WNT3A (0.1 μg/μL, 20 μL) recombinant protein mixture was injected into the injury sites daily for two consecutive days. TA muscles from control (n = 4) and RSPO3/WNT3A (n = 4) mice were harvested on seven dpi for subsequent analysis. **(B)** Representative immunofluorescence images of FABP4 (Cy3, red) and laminin (Alexa 488, green); nuclei were counterstained with DAPI (blue). Scale bar = 100 μm. **(C)** Violin plots showing the distribution of CTCF values among FABP4-positive cells from individual mice (n = 4 mice/group). Each violin represents 1 mouse. Solid and dotted white lines indicate the median and interquartile range, respectively. For all immunofluorescence staining quantifications, two sections from different depths of each muscle and 5–7 microscopic fields per section were analyzed for each mouse. More than 1,000 FABP4-positive cells were quantified per mouse, except for one control mouse, in which 768 cells were analyzed. **(D)** Group-level comparison of mean FABP4 CTCF values, showing individual mouse data points with the group mean ± SEM. Each dot represents the mean CTCF value from 1 mouse. **(E)** RT-qPCR analysis of adipogenic marker gene expression. Expression levels (normalized to *Actab*) were measured using independent biological triplicate from three control and three RSPO3/WNT3A-administered mice and are presented relative to the untreated control group. Bars indicate the mean ± SEM, and individual dots represent each mouse. Statistical significance was determined using unpaired two-tailed Student’s t-test. *p ≤ 0.05; **p ≤ 0.01; ***p ≤ 0.001; ****p ≤ 0.0001.

RSPO3/WNT3A-injected TA muscle showed significantly reduced FABP4 intensity compared to controls (Figures 6C,D; Supplementary Figure S5A). High-intensity FABP4-positive cells (top 10%) were particularly reduced (Supplementary Figure S5B). In accordance with this finding, PLIN1 immunofluorescence also showed a similar trend toward lower PLIN1+ adipocytes density in RSPO3/WNT3A-injected muscle group (Supplementary Figure S5C), although the difference was not statistically significant. Consistently, RT-qPCR revealed decreased expression of adipogenic genes in RSPO3/WNT3A-injected muscle relative to control muscle (Figure 6E). These results confirm that RSPO3/WNT3A inhibits ectopic adipogenic differentiation *in vivo*.

### 3.6 Reduced endogenous *Rspo2* and *Rspo3* expression does not enhance *in vivo* adipogenesis

To assess endogenous functions of *Rspo2* and *Rspo3* during glycerol-induced ectopic adipogenesis, considering the potential redundant functions of *Rspo2* and *Rspo3,* we used conditional *Rspo3* gene knockout mice (*Rspo3* ^*(fl/fl)*^;*CAGGCre-ER* ^*TM*^) combined with electroporation of *Rspo2* siRNA (Figure 7A). While *Rspo2* knockdown efficiency was ∼30–50%, conditional *Rspo3* knockout efficiency was >90% as determined by RT-qPCR (Figure 7B).

**FIGURE 7:**
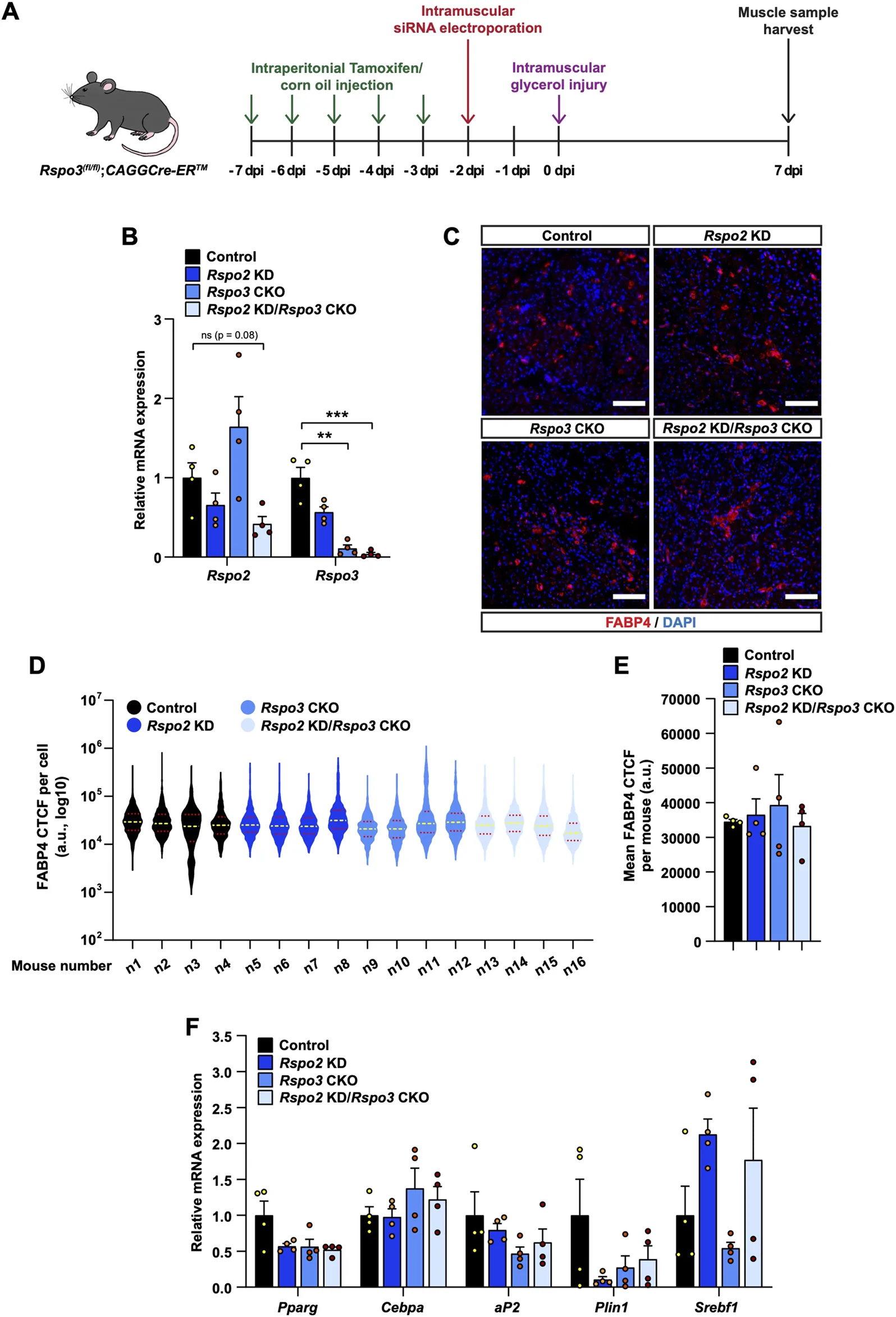
Combined *Rspo2* knockdown and *Rspo3* conditional knockout do not affect ectopic adipocyte accumulation in glycerol-injured TA muscle. **(A)** Experimental design for *Rspo2* siRNA electroporation and tamoxifen-induced *Rspo3* knockout in *Rspo3* ^*(fl/fl)*^;*CAGGCre-ER* ^*TM*^ mice. Tamoxifen or corn oil was administered intraperitoneally once daily for five consecutive days, followed by intramuscular siRNA injection and immediate electroporation into the TA muscle. Glycerol-induced TA muscle injury was performed 2 days after siRNA delivery, and TA muscles were harvested at 7 days post-injury (dpi). Four mice (n = 4) were used per experimental group for subsequent experiments: control (corn oil + non-target siRNA), *Rspo2* KD (corn oil + *Rspo2* siRNA), *Rspo3* CKO (tamoxifen + non-target siRNA), and *Rspo2* KD/*Rspo3* CKO (tamoxifen + *Rspo2* siRNA). **(B)** RT-qPCR analysis confirming the efficiency of *Rspo2* knockdown and *Rspo3* conditional knockout. Four independent RNA samples from each experimental group were analyzed, and relative mRNA expression was normalized to *Actab* (β-actin). Bars indicate the mean ± SEM, and individual dots represent each mouse. **(C)** Representative immunofluorescence images of FABP4 (Cy3, red); nuclei were counterstained with DAPI (blue). Scale bar = 100 μm. **(D)** Violin plots showing the distribution of CTCF values among FABP4-positive cells from individual mice (n = 4 mice/group). For all immunofluorescence staining quantifications, two sections from different depths of each muscle and 5–7 microscopic fields per section were analyzed for each mouse. Each violin represents 1 mouse. Yellow and red dotted lines indicate the median and interquartile range, respectively. More than 700 FABP4-positive cells were quantified per mouse, except for two control mice, in which 589 and 471 cells were analyzed, respectively. **(E)** Group-level comparison of mean FABP4 CTCF values, showing individual mouse data points with the group mean ± SEM. Each dot represents the mean CTCF value from 1 mouse. **(F)** RT-qPCR analysis of adipogenic marker gene expression. Expressions levels (normalized to *Actab*) are presented relative to the untreated control group. RNA samples in quadruplicate were used for each experimental group. Bars indicate mean ± SEM and individual dots represent each mouse. Statistical significance was determined using one-way ANOVA with Tukey’s *post hoc* test. *p ≤ 0.05; **p ≤ 0.01; ***p ≤ 0.001; ****p ≤ 0.0001.

We examined ectopic adipogenesis in TA muscle after glycerol injury in four groups were analyzed: control, *Rspo2* KD, *Rspo3* CKO, and *Rspo2* KD*;Rspo3* CKO. No significant differences in adipogenesis were observed as assessed by FABP4 CTCF and adipogenic marker gene expression in all testing groups (Figures 7C–F and S5D). Lack of effect in double KD/CKO may be due to suboptimal *Rspo2* KD. Therefore, the function of endogenous *Rspo2* and *Rspo3* is inconclusive, and *Rspo2;Rspo3* double CKO model may be required.

### 3.7 Transcriptomic profiling of RSPO3/WNT3A-treated muscle

To gain molecular insights into RSPO3/WNT3A inhibition on ectopic adipogenesis, we performed total RNA sequencing (RNA-seq) analysis on TA muscles collected from PBS- and RSPO3/WNT3A-injected mice after glycerol injury (7 dpi). RNA-seq analysis identified 1,122 upregulated and 1,270 downregulated genes (fold change >1.5, FDR ≤0.05) (Supplementary Figure S6A).

Pathway analysis using ENRICHR online platform revealed that downregulated genes were enriched in adipogenesis, PPAR signaling pathway, and oxidative phosphorylation (Figure 8A; Supplementary Figures S6B-D). The non-redundant gene list was generated by combining the gene sets for adipogenesis and PPAR signaling pathway. In a √FPKM heatmap, expression of key adipogenic genes including *Fabp4* (Shan et al., 2013), *Lpin1* (Koh et al., 2008; Un Nisa et al., 2023), and *Acsl1* (Jiang et al., 2024) were downregulated in RSPO3/WNT3A-injected group compared to control group (Figure 8B).

**FIGURE 8:**
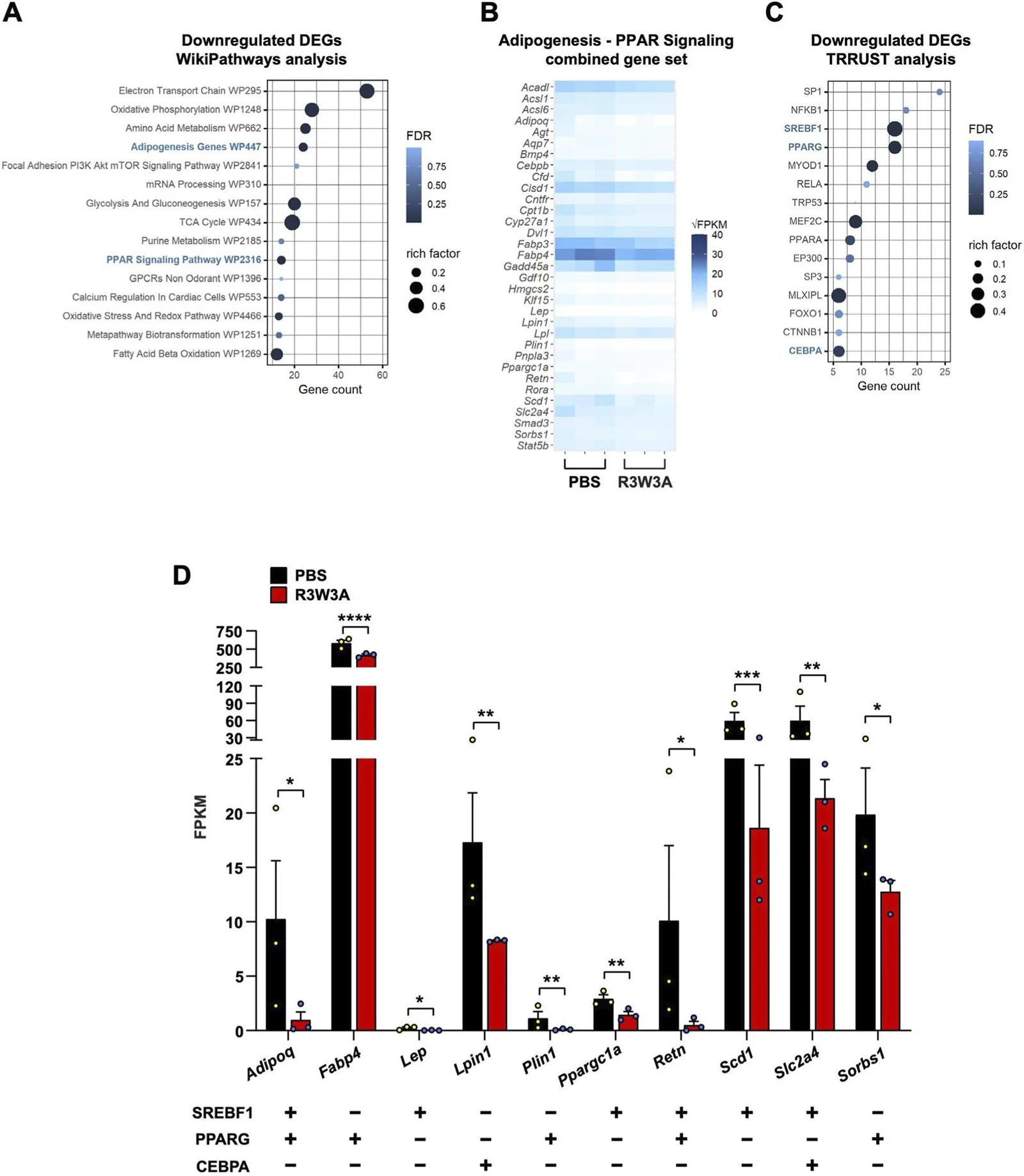
Transcriptome profiling reveals the downregulation of adipogenesis-associated genes in RSPO3/WNT3A-injected TA muscles compared to PBS-injected controls *in vivo*. **(A)** Bubble plot of WikiPathways (mouse; 2024 release) enrichment analysis on the downregulated DEGs in RSPO3/WNT3A-injected mouse muscle. Three mice were used for each of the control and RSPO3/WNT3A groups. Bubble size represents the rich factor, and color bar indicates the adjusted *p*-value (false discovery rate, FDR). The top 15 enriched terms ranked by gene count are displayed. **(B)** Expression heatmap of non-redundant DEGs identified in both adipogenesis genes and PPAR signaling pathway enriched terms. Color bar represents fragments per kilobase of transcript per million mapped reads (FPKM). **(C)** Bubble plot of TRRUST (mouse; 2019 release) transcription factor enrichment analysis on the downregulated DEGs in RSPO3/WNT3A-injected mouse muscle. Bubble size represents the rich factor, and color bar indicates the adjusted *p*-value (FDR). The top 15 enriched terms ranked by gene count are displayed and adipogenesis-related transcription factors are highlighted in bold blue. **(D)** Expression of overlapping genes identified from adipogenesis/PPAR pathways and transcription factors, including SREBF1, PPARG, and CEBPA in FPKM. Each gene is marked as a target of each transcription factor by +/−. Data are presented as mean ± standard deviation and individual dots represent each mouse. *q ≤ 0.05; **q ≤ 0.01; ***q ≤ 0.001; ****q ≤ 0.0001.

Transcription factor enrichment analysis was performed on downregulated genes utilizing TRRUST (Transcriptional Regulatory Relationships Unraveled by Sentence-based Text mining) database (Han et al., 2015). SREBF1, an early adipogenesis regulator (Ayala-Sumuano et al., 2011), and PPARG and CEBPA, two master adipogenic transcription factors (Gerstner et al., 2024), were identified (Figure 8C). We further selected the target genes of SREBP1, PPARG and CEBPA from the adipogenic non-redundant gene, respectively (Supplementary Figure S6E). Ten adipogenic genes, including *Adipoq*, *Retn*, *Fapb4*, and *Slc2a4*, were identified as targets of these three transcription factors (Figure 8D). MyoD and MEF2C, two major myogenic regulators, were also identified (Figure 8C), suggesting that RSPO3/WNT3A impacts on myogenesis during muscle regeneration.

Upregulated genes were enriched in focal adhesion, cell adhesion, and PI3K Akt mTOR signaling pathway (Figure 9A; Supplementary Figure S6B-D). Interestingly, heatmap analysis of the non-redundant genes contributing to this pathway revealed increased expression of several extracellular matrix (ECM)-associated genes in RSPO3/WNT3A-administered muscle samples, whereas most canonical focal adhesion genes remained largely affected (Figure 9B).

**FIGURE 9:**
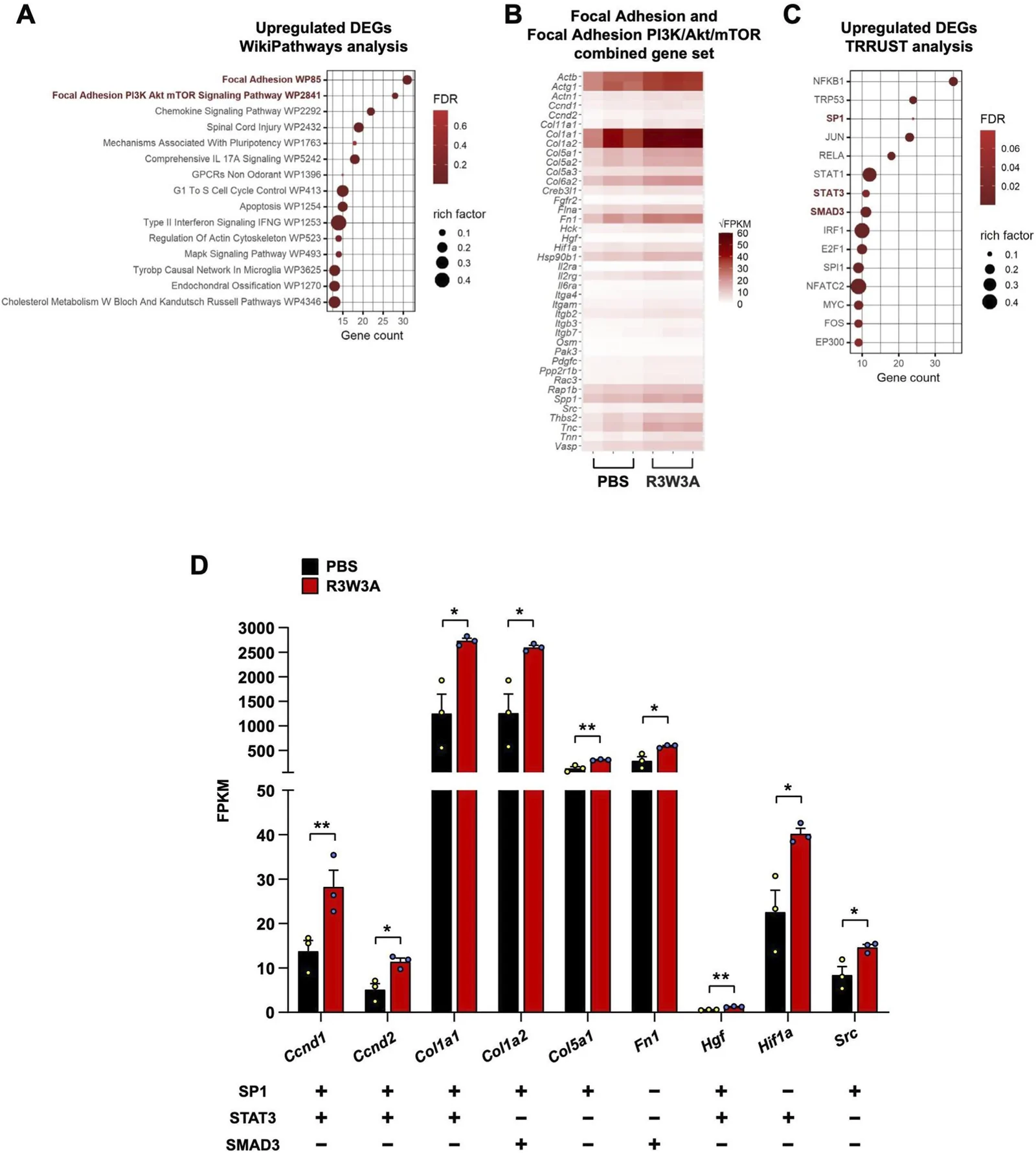
Transcriptome profiling reveals the upregulation of ECM- and fibrogenesis-associated genes in RSPO3/WNT3A-injected TA muscles compared to PBS-injected controls *in vivo*. **(A)** Bubble plot of WikiPathways enrichment analysis on the downregulated DEGs in RSPO3/WNT3A-injected mouse muscle. Bubble size represents the rich factor, and color bar indicates the adjusted *p*-value (FDR). The top 15 enriched terms ranked by gene count are displayed. **(B)** Expression heatmap of non-redundant DEGs identified in both focal adhesion and focal adhesion PI3/AKT/mTOR signaling pathway enriched terms. ECM-associated genes, including those encoding collagens and fibronectin, showed increased expression in RSPO3/WNT3A-injected muscles. Color bar represents FPKM. **(C)** Bubble plot of TRRUST factor enrichment analysis on the upregulated DEGs in RSPO3/WNT3A-injected mouse muscle. Bubble size represents the rich factor, and color bar indicates the adjusted *p*-value (FDR). The top 15 enriched terms ranked by gene count are displayed and transcription factors associated with ECM and fibrogenic phenotype are highlighted in bold red. **(D)** Expression of genes overlapping between the focal adhesion/PI3/AKT/mTOR pathways and targets of transcription factors SP1, STAT3, and SMAD4. Each gene is marked as a target of each transcription factor by +/−. Data are presented as mean ± standard deviation, and individual dots represent each mouse. *q ≤ 0.05; **q ≤ 0.01; ***q ≤ 0.001; ****q ≤ 0.0001.

Transcription factor analysis on the upregulated genes identified SP1, STAT3, and SMAD3 (Figure 9C). Among the non-redundant genes in focal adhesion, 9 genes were identified as targets of SP1, STAT3 and/or SMAD3 (Supplementary Figure S6F). ECM genes such as *Col1a1*, *Col1a2*, *Col5a1*, and *Fn1*, were identified (Figure 9D). Although RSPO3/WNT3A administration was expected to activate canonical WNT signaling, both up- and downregulated genes were not enriched in WNT signaling. Overall, these findings suggest that RSPO3/WNT3A suppresses adipogenesis by targeting key adipogenic transcriptional networks while concomitantly promoting the expression of ECM-associated gene expression involved in ECM remodeling or fibrogenic processes.

## 4 Discussion

In this study, we demonstrate that RSPO2 and RSPO3 function as critical regulators of adipogenic differentiation in fibro/adipogenic progenitors (FAPs), primarily through modulation of canonical WNT/β-catenin signaling. Our findings provide both *in vitro* and *in vivo* evidence that RSPO-mediated signaling suppresses adipogenesis and contributes to the regulation of intramuscular fat formation during muscle regeneration.

Our initial single-cell transcriptomic and ddPCR analyses revealed that *Rspo2* and *Rspo3* are two major members of the *Rspo* family expressed in FAPs, with *Rspo3* being the most abundant. The enrichment of *Rspo* expression in *Pdgfra-, Thy1-, and Ly6a*-positive FAP population suggests a cell type–specific role for RSPO signaling within the muscle stromal compartment. Although MuSCs expressed relatively low levels of these genes, the presence of RSPOs in FAPs points to a potential autocrine or paracrine regulatory mechanism within the muscle niche.

Functionally, we show that RSPO2 and RSPO3 significantly enhance the anti-adipogenic effects of WNT3A. While RSPOs alone exerted minimal effects, their combination with WNT3A robustly inhibited lipid accumulation and suppressed the expression of key adipogenic transcription factors, including PPARγ and C/EBPα. The function of both transcription factors is also regulated by their nuclear localization, without corresponding changes in total protein levels (Vasseur-Cognet and Lane, 1993; Ayers et al., 2007; Umemoto and Fujiki, 2012). It therefore remains as an intriguing question whether RSPOs alone or in combination with WNT together can alter the nuclear localization of these factors independently of change in total protein expression. Nevertheless, these results are consistent with the established role of RSPO proteins as potentiators of canonical WNT signaling (Jin and Yoon, 2012; Raslan and Yoon, 2019) and indicate that RSPO2/3 act to amplify WNT-dependent inhibition of adipogenesis in FAPs.

Our loss-of-function studies further support a physiological role for endogenous RSPO2 and RSPO3 in restraining adipogenic differentiation. Although individual knockdown of *Rspo2* or *Rspo3* in FAPs *in vitro* modestly increased adipogenesis, simultaneous knockdown produced a more pronounced effect, suggesting functional redundancy between these ligands. This redundancy is consistent with previous reports describing overlapping activities among RSPO family members in other tissues (Neufeld et al., 2012; Hagner et al., 2020; Dasgupta et al., 2021). Importantly, these findings imply that basal RSPO signaling contributes to maintaining FAPs in a non-adipogenic state under normal conditions.

Mechanistically, we demonstrate that the anti-adipogenic effects of RSPO3/WNT3A are dependent on β-catenin. Reduction of β-catenin levels partially rescued adipogenic differentiation in the presence of RSPO3 and WNT3A, whereas constitutive activation of β-catenin strongly suppressed adipogenesis. These results establish canonical WNT/β-catenin signaling as a central mediator of RSPO function in FAPs. Given that β-catenin activation is known to inhibit adipogenic commitment while promoting alternative lineage programs in mesenchymal stem cells and preadipocytes (Shang et al., 2007; Cawthorn et al., 2012; Grunberg et al., 2014), our findings align with a broader framework in which WNT signaling maintains progenitor cell identity and prevents adipocyte differentiation.

During adipogenic differentiation of FAPs, β-catenin expression decreased at an early stage and subsequently recovered at later stages of differentiation (Supplementary Figure S3A), suggesting a potential stage-dependent role of canonical WNT signaling. Gene knockdown of *Rspo2*, *Rspo3*, and *Ctnnb1* was performed immediately before the initiation of adipogenic differentiation, and the knockdown efficiency was assessed at an early stage. However, whether depletion of these genes persisted at later stages of adipogenesis was not determined. Previous studies have demonstrated that transient perturbation of canonical WNT signaling during the early stage of adipogenic differentiation can influence subsequent differentiation in other adipogenic cell contexts (Bennett et al., 2002; Park et al., 2019). Therefore, the early reduction in *Rspo2*, *Rspo3*, and *Ctnnb1* expression may contribute to the subsequent enhancement of FAP adipogenesis, making it difficult to determine the potential later-stage roles of canonical WNT signaling. Further studies specifically targeting the later stages of adipogenesis will be important to determine whether canonical WNT signaling has distinct stage-dependent functions during FAP adipogenesis.

Our *in vivo* experiments extend the *in vitro* findings to a physiologically relevant context. Administration of RSPO3 and WNT3A in a glycerol-induced muscle injury model significantly reduced ectopic adipogenesis, as evidenced by decreased FABP4 expression and downregulation of adipogenic genes. Although variation in FAP abundance and differentiation potential among different muscle groups is well documented (Lee et al., 2020), it is noteworthy that *in vitro* studies using whole-hindlimb-derived FAPs and *in vivo* studies using the TA muscle yielded similar conclusions, suggesting that muscle type-specific differences may not substantially affect the overall findings. These data indicate that RSPO/WNT signaling can modulate the regenerative microenvironment and limit pathological fat accumulation following muscle injury.

Transcriptomic profiling of RSPO3/WNT3A-adminstered glycerol-injured TA muscles provided further insight into the molecular mechanisms underlying RSPO3/WNT3A function. Downregulated gene sets were strongly enriched for adipogenesis and PPAR signaling pathways, with key transcription factors such as SREBF1, PPARG, and CEBPA identified as central nodes. The coordinated suppression of their downstream targets suggests that RSPO/WNT signaling interferes with the core transcriptional network driving adipocyte differentiation. Interestingly, oxidative phosphorylation pathways were also downregulated, consistent with the metabolic reprogramming that accompanies adipogenesis (Ryu et al., 2013).

Conversely, the increased expression of collagen and fibronectin genes, potentially regulated by SP1, STAT3, and SMAD3, suggests that RSPO3/WNT3A may promote an ECM-remodeling and fibrogenic transcriptional phenotype in FAPs. These findings raise the possibility that suppression of adipogenesis is accompanied by a reciprocal shift toward ECM production and fibrogenic processes. Consistent with this possibility, our preliminary results showed that both RSPO2 and RSPO3, in combination with WNT3A, increased COL1A1 and CTGF protein expression in FAPs, suggesting that RSPO/WNT3A signaling may promote a fibrogenic phenotype (Supplementary Figure S7). Further systematic studies are needed to define the functional relationship and potential interplay between adipogenic and fibrogenic differentiation of FAPs.

Notably, despite the expected activation of canonical WNT signaling, pathway enrichment analyses did not prominently identify WNT-related terms, especially in the upregulated genes. This may reflect both the transient nature of WNT target gene activation by negative feedback (MacDonald et al., 2009) and timing of muscle harvest after RSPO/WNT administration. Furthermore, because activation of WNT/β-catenin signaling primarily leads to transcription activation of the target genes, the pathways including adipogenesis that are identified in the downregulated genes are likely the secondary consequences. Alternatively, RSPO/WNT signaling may exert its effects through downstream transcriptional networks (de Winter and Nusse, 2021) that are more readily captured in pathway analyses.

*In vivo* functional studies result in uncertainty on endogenous function of *Rspo2* and *Rspo3* in FAP adipogenesis. *Rspo3* ablation did not markedly affect on adipogenesis, suggesting the functional redundancy of *Rspo2* as seen *in vitro*. Interestingly and unexpectedly, combined reduction of endogenous *Rspo2* and *Rspo3* did not significantly enhance adipogenesis *in vivo*. This discrepancy with *in vitro* results may reflect incomplete knockdown of *Rspo2*, compensatory mechanisms from other RSPO family members, or the complexity of the *in vivo* niche, where multiple signaling pathways converge to regulate FAP fate (Giuliani et al., 2022). In addition, because we only analyzed the samples at 7 dpi, *Rspo2* knockdown effects during earlier stage may have been missed. While *Rspo2*/*Rspo3* functions in FAP adipogenesis remain inconclusive, future studies utilizing genetic *Rspo2*/*Rspo3* double knockout models will be necessary to fully elucidate the contribution of endogenous RSPO signaling *in vivo*.

In summary, our study identifies RSPO2 and RSPO3 as important modulators of FAP fate, acting through canonical WNT/β-catenin signaling to suppress adipogenesis and regulate muscle regeneration. These findings have potential implications for conditions characterized by pathological intramuscular fat accumulation, such as muscular dystrophy (Cardone et al., 2023), aging-related sarcopenia (Moratal et al., 2019), and metabolic disorders (Mogi et al., 2016). Targeting RSPO/WNT signaling pathways may represent a promising therapeutic strategy to control FAP differentiation and improve muscle tissue quality. Further investigation into the balance between adipogenic and fibrogenic outcomes will be essential for translating these findings into clinical applications.

## Data Availability

The original contributions presented in the study are publicly available. This data can be found in the NCBI Gene Expression Omnibus (GEO) repository with accession number: GSE349326.
