# Supplementary material for: R-spondins suppress adipogenic differentiation in fibro-adipogenic progenitors via autocrine WNT/β-catenin signaling
Source: Front Cell Dev Biol. 2026 Oct 16;14:1942626. doi: 10.3389/fcell.2026.1942626 (PMC13719486; doi:10.3389/fcell.2026.1942626)
Supplement: Supplementary file 1 [file DataSheet1.docx]

**SUPPLEMENTARY FIGURE LEGENDS**

**Figure S1.**

**Additional data demonstrating the inhibitory effect of RSPO2/WNT3A and RSPO3/WNT3A treatments on FAP adipogenesis.** (A and B) Oil Red O (ORO) staining analysis at AD7 following RSPO2/WNT3A (A) or RSPO3/WNT3A (B) treatment, including representative images (scale bar = 200 μm) and corresponding absorbance quantification. ORO quantification was performed using three independent biological replicates (n=3) and is presented as mean ± SEM of absorbance values at 490 nm after baseline subtraction using AD0 values. (C and D) Western blot analysis of adipogenic marker expression for the same treatment groups: RSPO2/WNT3A (C) and RSPO3/WNT3A (D) at AD6. Immunoblots from two independent biological replicates (n=2) are shown side by side. Densitometric quantification was performed for each immunoblot, and the resulting values are presented as numbers below the corresponding lanes, indicating the relative fold change compared with the control from the same immunoblot (control value set to 1.00), after normalization to Vinculin. All bar graphs include individual dots representing each biological replicate. Statistical significance was determined using one-way ANOVA with Tukey’s post hoc test. **p* ≤ 0.05; ***p* ≤ 0.01; ****p* ≤ 0.001; *****p* ≤ 0.0001.

**Figure S2**

**Independent replicate immunoblots corresponding to the main figures.** (A and B) Independent replicate immunoblots showing AD3 adipogenic marker expression following RSPO2/WNT3A (A) and RSPO3/WNT3A (B) treatment at AD3, corresponding to Figure 2D and 2E, respectively. (C) Independent replicate immunoblots showing adipogenic marker expression for *Rspo2*- and *Rspo3*- knockdown cells at AD3 corresponding to Figure 3D. (D) Independent replicate immunoblots showing β-catenin knockdown efficiency and adipogenic marker expression in *Ctnnb1*-knockdown FAPs following RSPO3/WNT3A treatment at AD3, corresponding to Figure 4C. (E) Independent replicate immunoblots showing β-catenin and adipogenic marker expression in 4-OHT treated FAPs at AD3, corresponding to Figure 5D. Numbers below the corresponding lanes indicate relative protein levels normalized to the respective loading control, with the control value from the same immunoblot set to 1.00.

**Figure S3**

**Additional data supporting the roles of endogenous *Rspo2* and *Rspo3* in suppressing FAP adipogenic differentiation.** (A) Representative images (scale bar = 200 μm) and quantification of ORO staining analysis at AD7. Absorbances at 490 nm were measured using three independent biological replicates (n=3) and are presented after baseline subtraction using AD0 values. (B) Western blot analysis of adipogenic marker expression in *Rspo2* and *Rspo3* knockdown cells at AD6. Immunoblots from two independent biological replicates (n=2) are shown side by side. Corresponding densitometric quantification was performed for each immunoblot, and the resulting values are presented as numbers below the corresponding lanes, indicating the relative fold change compared with the control from the same immunoblot (control value set to 1.00), after normalization to β-Actin. All bar graphs include individual dots representing each biological replicate. Statistical significance was determined using one-way ANOVA with Tukey’s post hoc test. **p* ≤ 0.05; ***p* ≤ 0.01; ****p* ≤ 0.001; *****p* ≤ 0.0001.

**Figure S4**

**Additional data supporting that the inhibitory effect of RSPO3/WNT3A on FAP adipogenesis is mediated through canonical WNT signaling in a β-Catenin-dependent manner.** (A) Western blot analysis showing the expression of β-Catenin and adipogenic markers during FAP adipogenic differentiation up to AD10. (B) Western blot analysis of β-Catenin expression following RSPO3/WNT3A treatment at AD3. Immunoblots from two independent biological replicates (n=2) are shown side by side. Corresponding densitometric quantification was performed for each immunoblot, and the resulting values are presented as numbers below the corresponding lanes, indicating the relative fold change compared with the control from the same immunoblot (control value set to 1.00), after normalization to β-Actin. (C) Western blot analysis showing β-Catenin siRNA knockdown efficiency and adipogenic marker expression at AD6. Immunoblots from two independent biological replicates (n=2) are shown side by side. Corresponding densitometric quantification was performed for each immunoblot, and the resulting values are presented as numbers below the corresponding lanes, indicating the relative fold change compared with the control from the same immunoblot (control value set to 1.00), after normalization to β-Actin expression. All bar graphs include individual dots representing each biological replicate. Statistical significance was determined using one-way ANOVA with Tukey’s post hoc test. **p* ≤ 0.05; ***p* ≤ 0.01; ****p* ≤ 0.001; *****p* ≤ 0.0001.

**Figure S5**

**Additional data corroborating the impact of RSPO3/WNT3A administration and *Rspo2/3* gene depletion in glycerol injury in TA muscle.** (A) Distribution of cell-level FABP4 CTCF values shown as scatter plots for individual mice (4 controls and 4 RSPO3/WNT3A-injected). Each dot represents a single cell, and horizontal white lines indicate the mean value per mouse. (B) Distribution of cells with low (˂P10), intermediate (P10-P90), and high (>P90) FABP4 CTCF categories are defined by pooled percentile thresholds (P10 = 5252.88 a.u.; P90 = 37325.7 a.u.). (C) Representative PLIN1 immunofluorescence images showing PLIN1 expression (scale bar = 100 μm) and corresponding quantification of PLIN1-positive adipocyte density within the injured area. For (B) and (C), data are presented as mean ± SEM, with individual dots representing individual mice. (D) Distribution of cell-level FABP4 CTCF values shown as scatter plots for individual mice (control, *Rspo2* KD, *Rspo3* CKO, and *Rspo2* KD;*Rspo3* CKO). Each dot represents a single cell, and horizontal white lines indicate the mean value per mouse. Statistical significance was determined using unpaired two-tailed Student’s t-test. **p* ≤ 0.05; ***p* ≤ 0.01; ****p* ≤ 0.001; *****p* ≤ 0.0001.

**Figure S6**

**Multi-database enrichment analysis of differentially expressed genes (DEGs) in RSPO3/WNT3A-injected mouse muscles (n=3) compared to PBS-injected controls (n=3).** (A) Volcano plot showing DEGs, with downregulated genes in blue and upregulated genes in red. Genes with less than 1.5-fold change and a false discovery rate (FDR) greater than 0.05 are indicated in black and separated by dotted threshold lines. (B-D) Bubble plots of pathway enrichment analyses for Reactome (B), KEGG (C), and Gene Ontology-Biological processes (GO-BP) (D). Downregulated and upregulated DEG sets are shown in blue and red, respectively. Bubble size represents the rich factor, and color bar indicates the adjusted *p*-value (FDR). The top 15 enriched terms ranked by gene count are displayed. (E and F) Venn diagrams showing overlapping gene numbers identified from Wikipathway and TRRUST enrichment analyses. (E) Common downregulated DEGs enriched in both the adipogenesis/PPAR pathway and transcriptional targets of SREBF1, PPARG, or CEBPA. (F) Common upregulated DEGs enriched in both the focal adhesion/PI3/AKT/mTOR pathways and transcriptional targets of SP1, STAT3, or SMAD4.

**Figure S7**

**RSPO3/WNT3A treatment promotes fibrogenic phenotypes in FAPs.** (A) Experimental design for investigating the effects of RSPO3/WNT3A treatments during FAP fibrogenic differentiation. FAPs were cultured in growth medium for 2 days and subsequently maintained under spontaneous fibrogenic differentiation in basal medium supplemented with RSPO3 (50 ng/ml), and WNT3A (10 ng/ml) for 3 days. (B) Immunofluorescence analysis following RSPO3/WNT3A treatment. Representative images of COL1A1 (scale bar = 100 μm) are shown and quantification of COL1A1+ cell numbers. COL1A1+ cell numbers are presented as mean ± SEM. Samples were prepared as three independent biological replicates (n=3). All bar graphs include individual dots representing each biological replicate. Statistical significance was determined using one-way ANOVA with Tukey’s post hoc test. **p* ≤ 0.05; ***p* ≤ 0.01; ****p* ≤ 0.001; *****p* ≤ 0.0001. (C and D) Western blot analysis of fibrogenic marker expression in FAPs treated with RSPO2 (C) and RSPO3(D), in combination with WNT3A. Densitometric quantification was performed for each immunoblot, and the resulting values are presented as numbers below the corresponding lanes, indicating the relative fold change compared with the control from the same immunoblot (control value set to 1.00), after normalization to β-Actin.
