# Supplementary material for: R-spondins suppress adipogenic differentiation in fibro-adipogenic progenitors via autocrine WNT/β-catenin signaling
Source: Front Cell Dev Biol. 2026 Oct 16;14:1942626. doi: 10.3389/fcell.2026.1942626 (PMC13719486; doi:10.3389/fcell.2026.1942626)
Supplement: Supplementary file 2 [file Table1.docx]

Table S1. Primer sequences used for ddPCR and RT-qPCR in materials and methods

| **Reagent** | **Source** | **Identifier** |
| --- | --- | --- |
| *Actb* qPCR primer (Forward) | Cosmogenetech | 5’-ATGGAGGGGAATACAGCCC-3’ |
| *Actb* qPCR primer (Reverse) | Cosmogenetech | 5’-TTCTTTGCAGCTCCTTCGTT-3’ |
| *Rspo1* qPCR primer (Forward) | Cosmogenetech | 5’-AATGCATCAAATGCAAGATCG-3’ |
| *Rspo1* qPCR primer (Reverse) | Cosmogenetech | 5’-TCATTTCACATTGTGCAGGAC-3’ |
| *Rspo2* qPCR primer (Forward) | Cosmogenetech | 5’-CCAAGGCAACCGATGGAGAC-3’ |
| *Rspo2* qPCR primer (Reverse) | Cosmogenetech | 5’-TCGGCTGCAACCATTGTCC-3’ |
| *Rspo3* qPCR primer (Forward) | Cosmogenetech | 5’-ATGCACTTGCGACTGATTTCT-3’ |
| *Rspo3* qPCR primer (Reverse) | Cosmogenetech | 5’-GCAGCCTTGACTGACATTAGGAT-3’ |
| *Rspo4* qPCR primer (Forward) | Cosmogenetech | 5’-CTCGCCCTGTACCGAAGGA-3’ |
| *Rspo4* qPCR primer (Reverse) | Cosmogenetech | 5’-CACTTGCCGTACTGACGGATG-3’ |
| *Pparg* qPCR primer (Forward) | Cosmogenetech | 5’-TCAGCTCTGTGGACCTCTCC-3’ |
| *Pparg* qPCR primer (Reverse) | Cosmogenetech | 5’-ACCCTTGCATCCTTCACAAG-3’ |
| *Cebpa* qPCR primer (Forward) | Cosmogenetech | 5’-CAAGAACAGCAACGAGTACCG-3’ |
| *Cebpa* qPCR primer (Reverse) | Cosmogenetech | 5’-GTCACTGGTCAACTCCAGCAC-3’ |
| *aP2* qPCR primer (Forward) | Cosmogenetech | 5’-GATGCCTTTGTGGGAACCT-3’ |
| *aP2* qPCR primer (Reverse) | Cosmogenetech | 5’-CTGTCGTCTGCGGTGATTT-3’ |
| *Plin1* qPCR primer (Forward) | Cosmogenetech | 5’-GTGCAATGCCTATGAGAAGGGTGTAC-3’ |
| *Plin1* qPCR primer (Reverse) | Cosmogenetech | 5’-GTAGAGATGGTGCCCTTCAGTTCAGA-3’ |
| *Srebf1* qPCR primer (Forward) | Cosmogenetech | 5’-GCAGCCACCATCTAGCCTG-3’ |
| *Srebf1* qPCR primer (Reverse) | Cosmogenetech | 5’-CAGCAGTGAGTCTGCCTTGAT-3’ |

Table S2. siRNA sequences used for gene knockdown experiments.

| **Target gene** | **Source** | **Target sequence** |
| --- | --- | --- |
| *Rspo2* (siRNA-1) | Dharmacon | 5’-AGACGCAAUAAGCGAGCUA-3’ |
| *Rspo2* (siRNA-2) | Dharmacon | 5’-GUUGGUCAUUGGAGCGAAU-3’ |
| *Rspo2* (siRNA-3) | Dharmacon | 5’-GGGCUUGAUUCUAACCGUA-3’ |
| *Rspo2* (siRNA-4) | Dharmacon | 5’-GGACAAUGGUUGCAGCCGA-3’ |
| *Rspo3* (siRNA-1) | Dharmacon | 5’-CCAAGUGGAUAUUACGGAA-3’ |
| *Rspo3* (siRNA-2) | Dharmacon | 5’-UGUGCAACGUGUUCAGAUU-3’ |
| *Rspo3* (siRNA-3) | Dharmacon | 5’-UCAUGUAAGCCCAGACUGU-3’ |
| *Rspo3* (siRNA-4) | Dharmacon | 5’-GUUAGAAGCCAACAAUCAU-3’ |
| *Ctnnb1* (siRNA-1) | Dharmacon | 5’-GUGAAAUUCUUGGCUAUUA-3’ |
| *Ctnnb1* (siRNA-2) | Dharmacon | 5’-GCGCUUGGCUGAACCAUCA-3’ |
| *Ctnnb1* (siRNA-3) | Dharmacon | 5’-CAGCAAAUCAUGCGCCCUU-3’ |
| *Ctnnb1* (siRNA-4) | Dharmacon | 5’-AAGCUGACCUGAUGGAGUU-3’ |

Table S3. Marker genes used for scRNA-seq cell-type annotation

| **Cell population / cluster** | **Marker genes used for annotation** |
| --- | --- |
| Endothelial cells | *Pecam1*, *Cdh5* |
| Fibro-adipogenic progenitors (FAPs) | *Pdgfra*, *Thy1*, *Ly6a* |
| Myofibroblasts | *Tagln*, *Myl9*, *Acta2*, *Dcn, Col1a1* |
| Smooth muscle cells | *Acta2*, *Myh11* |
| B and T cells | *Cd19*, *Ms4a1* (B cells); *Cd28*, *Cd3g* (T cells) |
| Muscle stem cells (MuSCs) | *Pax7*, *Myf5* |
| Monocytes | *Itgam*, *Sell*; relatively low/absent *Adgre1* |
| Macrophages | *Cd68*, *Adgre1* |
| Glial cells | *Mpz*, *Sox10*, *Dhh* |
| Muscle-lineage related cells | *Ckm*, *Acta1*, *Myh4* |
